# Comparative efficacy and safety of molecular targeted agents combined with transarterial chemoembolization in the treatment of unresectable hepatocellular carcinoma: a network meta-analysis

**DOI:** 10.3389/fonc.2023.1179431

**Published:** 2023-05-17

**Authors:** Jiaye Long, Baoxiang Chen, Zhaohui Liu

**Affiliations:** ^1^ Department of Interventional Radiology, Inner Mongolia Forestry General Hospital, The Second Clinical Medical School of Inner Mongolia University for The Nationalities, Yakeshi, Inner Mongolia, China; ^2^ Department of Urology, Inner Mongolia Forestry General Hospital, The Second Clinical Medical School of Inner Mongolia University for The Nationalities, Yakeshi, Inner Mongolia, China

**Keywords:** transarterial chemoembolization, molecular targeted agents, hepatocellular carcinoma, network meta-analysis, systematic review

## Abstract

**Objective:**

At present, several molecular targeted agents(MTAs) combined with transarterial chemoembolization (TACE) have been employed to treat unresectable hepatocellular carcinoma (HCC). In this meta-analysis, we compared the efficacy and safety of different MTAs combined with TACE to enable effective decision-making for the clinical treatment of unresectable HCC.

**Methods:**

Pubmed, Web of Science, EMBASE, and Cochrane Library were retrieved to evaluate the efficacy and safety of different MTAs combined with TACE in cohort studies and randomized controlled trials. The hazard ratios and 95% confidence intervals (CIs) were calculated to investigate the impact of various therapies on overall survival (OS) and progression-free survival. However, the objective response rate (ORR), disease control rate (DCR), adverse events (AEs), and ≥grade-3 adverse events (≥G3-AEs) were calculated using odd ratios and 95% CIs. The node-splitting approach was used to test the heterogeneity. The funnel plot was utilized to analyze the publication bias. Additionally, according to the ranking plots, we ranked various treatments.

**Results:**

A total of 45 studies involving 10,774 patients with 8 treatment strategies were included in our network meta-analysis. Our network meta-analysis showed that apatinib+TACE provided the highest OS (62.2%), ORR (44.7%), and DCR (45.6%), while and lenvatinib+TACE offered the best PFS (78.9%). Besides, there was no statistically significant difference in AEs and ≥G3-AEs among treatment options.

**Conclusion:**

Apatinib+TACE demonstrated the best OS, ORR, and DCR with no additional AEs and ≥G3-AEs. Therefore, for the treatment scheme of MTAs combined with TACE, apatinib+TACE may be the best option for patients with unresectable HCC.

**Systematic review registration:**

https://www.crd.york.ac.uk/PROSPERO/, identifier CRD42023388609.

## 1 Introduction

As one of the most prevalent kinds of cancer, primary liver cancer (PLC) incidence rate and mortality rank sixth and third globally, respectively (1). Hepatocellular carcinoma (HCC) accounts for 75% to 95% of PLC cases (1). Features of HCC include insidious onset, lengthy latency, and swift progression. Patients are frequently diagnosed at an advanced stage, making them miss out on the best opportunity for surgery (2). The diagnosis of advanced HCC is found in patients who do not follow the recommended monitoring plan according to the guidelines. According to the guidelines, they should better accept different treatment strategies based on the number and size of HCC. Meanwhile, the median survival of advanced HCC is under one year, making it a significant global health problem (3).

For unresectable HCC (uHCC), the available treatment options mainly include transarterial chemoembolization (TACE), transarterial radioembolization (TARE), liver transplantation, stereotactic body radiation therapy (SBRT), targeted therapy, and immunotherapy (4). Due to its safety, efficacy, minimally invasive nature, and repeatability, TACE has been included as a first-line treatment in the non-radical treatment of HCC which cannot be surgically resected (5). TACE is mainly used to achieve the therapeutic purpose by injecting chemotherapy drugs into the tumor supply arteries and then blocking the above arteries with embolic materials. However, after TACE, the local hypoxia of the tumor blood supply artery will disturb the tumor microenvironment, leading to the upward regulation of hypoxia-inducible factor-1 (HIF-1), which upregulates vascular endothelial growth factor receptor (VEGFR) and platelet-derived growth factor receptor (PDGFR), further increasing tumor angiogenesis (6). Tumor neovascularization forms collateral circulation with other intrahepatic vessels, causing local recurrence and metastasis (7).

Over the past decades, the molecular mechanism of the onset and development of liver cancer has gradually become known through the continued exploration of molecular cell biology. The above progress provides a theoretical basis for the emergence of more molecular targeted agents (MTAs) which inhibit anomalous molecular targets (8). Tumor angiogenesis produced by ascending regulation of VEGFR and PDGFR is the primary cause of tumor spread and relapse following TACE. MTAs can inhibit the PFGF and VEGFR pathways, preventing tumor neovascularization. Meanwhile, molecular targeted therapy reduces tumor growth and differentiation by disrupting tumor signal transduction pathways, resulting in apoptosis and destruction of tumor cells. The Wnt/β-Catenin pathway, Ras/Raf/MAPK pathway, PI3/AKT/mTOR pathway, JAK/STAT pathway, Ubiquitin Proteasome pathway, and IGF1/IGF1R pathway are the principal targeted pathways for the therapy of HCC (8). Growth factors, signaling molecules, cyclins, apoptotic regulators, and chemicals that encourage angiogenesis in the route are among the compounds that targeted medications target (9). Different MTAs act on various transduction pathways, depending on the targets they are meant to affect. By obstructing signals that encourage cancer cell development, disrupting the control of the cell cycle, or inducing cell death, MTAs destroy cancer cells (10). Both TACE and MTAs have anti-tumor properties. At the same time, MTAs can reverse the tumor recurrence and metastasis caused by TACE treatment, which promotes tumor angiogenesis. Consequently, there is an increasing trend in clinical practice to combine TACE and MTAs to treat uHCC.

As more and more MTAs arrive on the market, so does the number of MTAs that TACE can jointly choose. However, due to the lack of head-to-head comparison of TACE in combination with MTAs, the ideal strategy for TACE in combination with MTA still needs to be discovered. Consequently, a network meta-analysis (NMA) was performed to compare the efficacy and safety of various MTAs combined with TACE.

## 2 Materials and methods

### 2.1 Protocol and registration

This NMA was registered in PROSPERO (CRD42023388609). Additionally, the study was conducted in strict adherence to the Preferred Reporting Items for Systematic Reviews and Meta-Analyses (PRISMA) guidelines.

### 2.2 Literature search strategy

We systematically searched Pubmed, Web of Science, EMBASE, and Cochrane Library from the date of establishment to January 4, 2023. The paramount search terms were “liver neoplasms”, “chemoembolization, therapeutic”, “sorafenib”, “sunitinib”, “brivanib”, “anlotinib”, “apatinib”, “orantinib”, “lenvatinib” along with their synonyms. The detailed search strategy is outlined in **Supplementary Table 1**.

### 2.3 Study inclusion and exclusion criteria

Studies were included in this NMA if they met the following inclusion criteria: (a) Patients: adults who were at least 18 years old diagnosed with uHCC; uHCC patients did not receive systematic treatment prior to receiving MTA combined with TACE or TACE alone; no additional treatment was administered during the studies, including radiofrequency ablation, percutaneous ethanol injection or iodine-125 seed implantation. (b) Intervention: TACE as monotherapy therapy or in combination with several MTAs. (c) Comparison: studies that compared the outcomes of various interventions in treating uHCC. (d) Outcomes: efficacy indicators included overall survival (OS), progression-free survival (PFS), objective response rate (ORR), and disease control rate (DCR); safety indicators included the incidence of adverse events (AEs) and ≥grade-3 adverse events (≥G3-AEs). (e) Study design: randomized controlled trials (RCTs) and cohort studies.

The following studies were eliminated from this NMA: case reports, reviews, case-control studies, editorials, and studies with insufficient data.

### 2.4 Data extraction and quality assessment

After determining the RCTs and cohort studies to be included in this study, two researchers (BC and ZL) independently extracted data. Any differences were resolved by a third researcher (JL). The following data were extracted: first author, publication year, region, treatment measures, sample size, gender, age, and disease characteristics.

We applied two quality evaluation tools to evaluate two types of studies. For cohort studies, we applied the Newcastle-Ottawa scale, which evaluated cohort studies through eight items. The eight items mentioned above consisted of the representativeness of the exposure cohort, the selection of the non-exposed cohort, the determination of the exposure, the absence of the disease to be studied at the beginning of the study, the comparability of the exposure cohort and the non-exposed cohort, the measurement method of the results, whether the follow-up time was long enough and the integrity of the follow-up. Apart from the item of comparability between exposed and non-exposed cohorts, which could be rated up to two stars, other items could be rated up to one star, with a total score of nine stars. For RCTs, the Cochrane’s Risk of Bias Tool, recommended by the Cochrane Handbook, was used to investigate sources of bias from seven dimensions. The seven dimensions were described in terms of six aspects, namely, selection bias, implementation bias, measurement bias, follow-up bias, reporting bias, and other biases. Each dimension was judged and divided by low, high, and unclear risk of bias.

### 2.5 Statistical analysis

R version 3.6.1 and StataMP 14.0 were used to analyze relevant data. We conducted a Bayesian NMA employing a random effect model to compare directly or indirectly the efficacy and safety of each treatment included in the study. To obtain the posterior distribution, we established three independent Markov chains for each outcome measure. The number of iterations per chain was set at 50,000, with the first 5,000 being considered burn-in samples. The model’s convergence was assessed employing Brooks-Gelman-Rubin plots and trace plots.

For OS and PFS, the pooled hazard ratio (HR) and 95% confidence intervals (CIs) were used for comparison. For ORR, DCR, AEs, and ≥G3-AEs, the pooled odds ratio (OR) and 95% CIs were used for comparison. We extracted data from Kaplan-Meier plots for those studies that did not offer HR values utilizing Engauge Digitizer version 11.3 software. The ranking probability was used to evaluate the ranking of each treatment measure. The node-splitting approach was used to determine if direct or indirect comparisons were coherent. Funnel plots were used to assess whether the included study had publication bias. If the funnel plot was symmetrical, it indicated no publication bias. Otherwise, there may be publication bias. Two-tailed P<0.05 was deemed statistically significant.

## 3 Results

### 3.1 Search results and quality assessment

**Figure 1:**
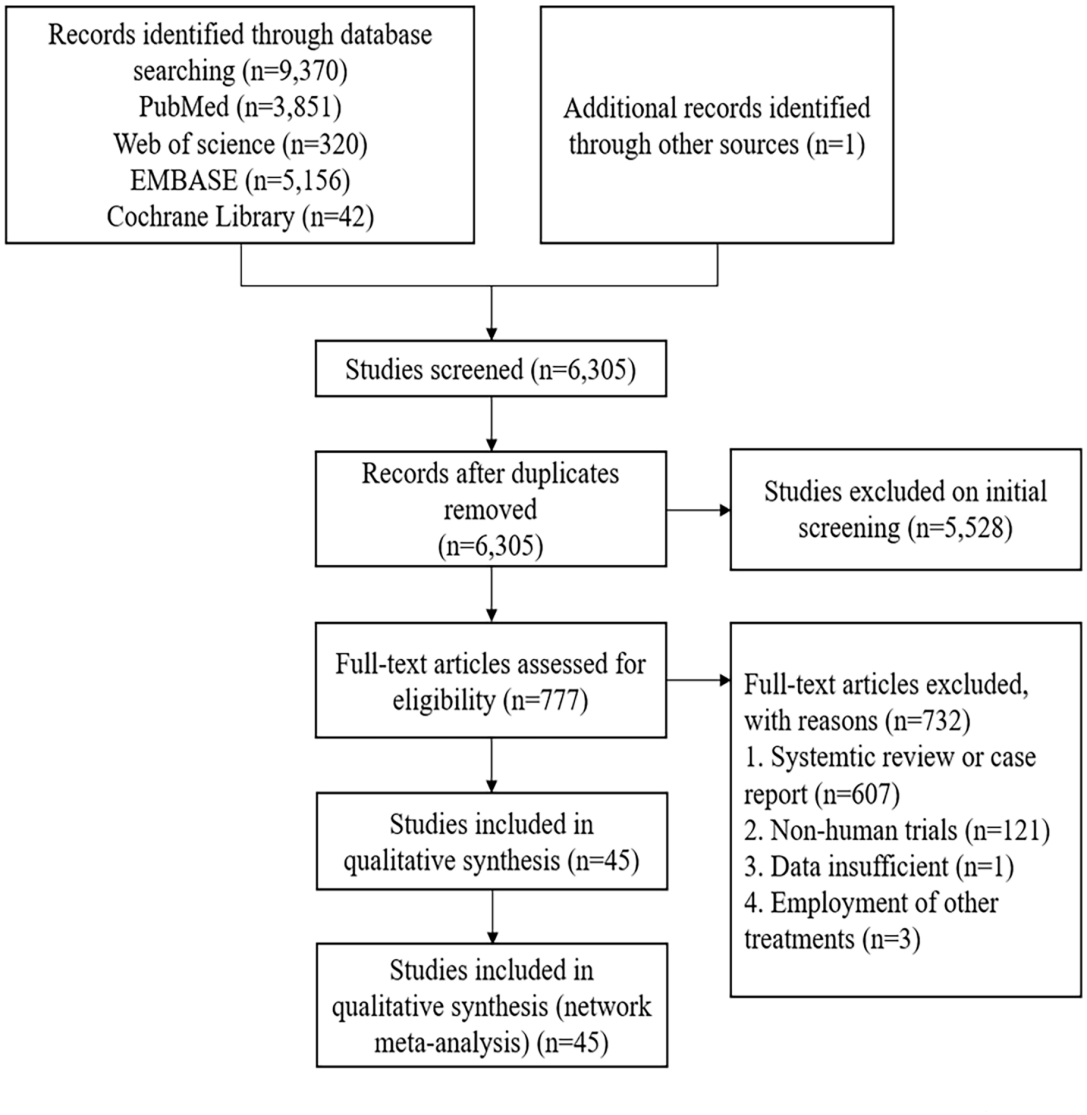
Flow chart of the study screening process.

In our selected database, 9,370 studies were initially identified, and another study was obtained through other means. After removing 2,966 duplicate articles, 6,305 articles were abstracted and screened. Following the preliminary screening, 777 articles met the evaluation criteria. Subsequently, after excluding 607 systematic reviews or case reports, 121 non-human trials, 1 article with incomplete data, and 3 articles receiving other treatments, a total of 10 RCTs (11–20) and 35 cohort studies (21–55) were included for NMA. The literature screening process is illustrated in **Figure 1**.

In our NMA, a total of 7 MTAs+TACE treatment schemes were included, namely: sorafenib+TACE (Sora+TACE), lenvatinib+TACE (Lenv+TACE), sunitinib+TACE (Suni+TACE), brivanib+TACE (Briv+TACE), anlotinib+TACE (Anlo+TACE), apatinib+TACE (Apat+TACE) and orantinib+TACE (Oran+TACE). There were 10,774 HCC patients in our 45 included studies. Among them, 19 studies (11–14, 21–35) were about the comparison of Sora+TACE and TACE monotherapy, 3 studies (36–38) were about the comparison of Lenv+TACE and TACE monotherapy, 11 studies (15, 39–48) were about the comparison of Apat+TACE and TACE monotherapy, 1 studies (49) was about the comparison of Anlo+TACE and TACE monotherapy, 1 study (16) was about the comparison of Briv+TACE and TACE monotherapy, and 2 studies (17, 50) were about the comparison of Suni+TACE and TACE monotherapy, and 3 studies (18–20) were about the comparison of Oran+TACE and TACE monotherapy. Moreover, there were 2 studies (51, 52) on the comparison of Lenv+TACE and Sora+TACE, 2 studies (53, 54) on the comparison of Sora+TACE and Apat+TACE, and 1 study (55) on the comparison of Suni+TACE and Sora+TACE. In the included studies, the patient count was between 42 and 1,719. The age of patients varied between 18 and 87 years. The characteristics of the included study are shown in **Supplementary Table 2**. The quality evaluation of the included literature is shown in **Supplementary Table 3**.

### 3.2 Overall survival

**Figure 2:**
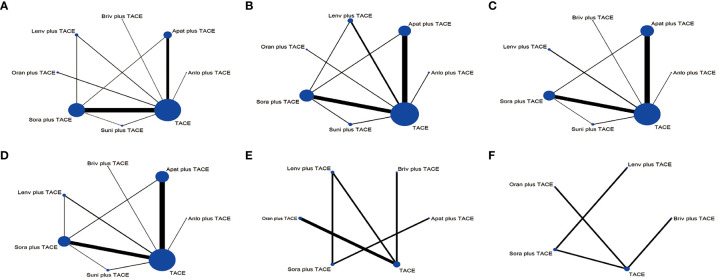
Network plots of the comparisons for the network meta-analysis. **(A)** Overall survival. **(B)** Progression-free survival. **(C)** Objective response rate. **(D)** Disease control rate. **(E)** ≥Grade-3 adverse events. **(F)** Adverse events. The size of the circle is proportional to the number of studies. The width of the line is proportional to the study of direct comparison. Lenv plus TACE, lenvatinib+TACE; Briv plus TACE, brivanib+TACE; Apat plus TACE, apatinib+TACE; Anlo plus TACE, anlotinib+TACE; Suni plus TACE, sunitinib+TACE; Sora plus TACE, sorafenib+TACE; Oran plus TACE, Orantinib+TACE.

**Figure 3:**
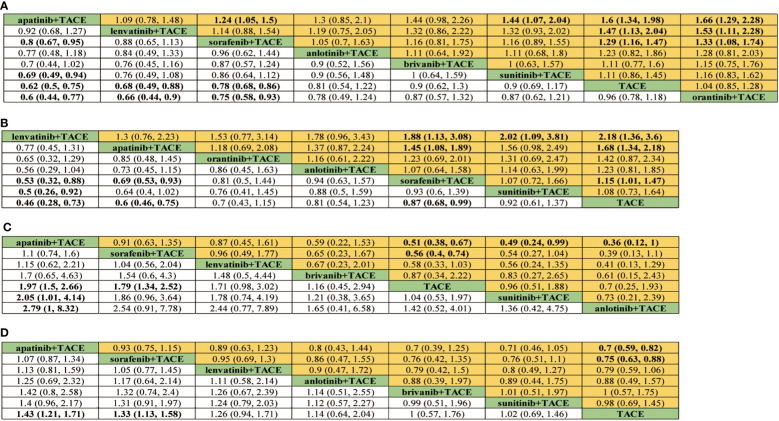
Pooled efficacy indicators estimates of network meta-analysis. **(A)** Pooled hazard ratios (95% confidence intervals) of overall survival. **(B)** Pooled hazard ratios (95% confidence intervals) of progression-free survival. **(C)** Pooled odds ratios (95% confidence intervals) for objective response rate. **(D)** Pooled odds ratios (95% confidence intervals) for disease control rate.

**Figure 4:**
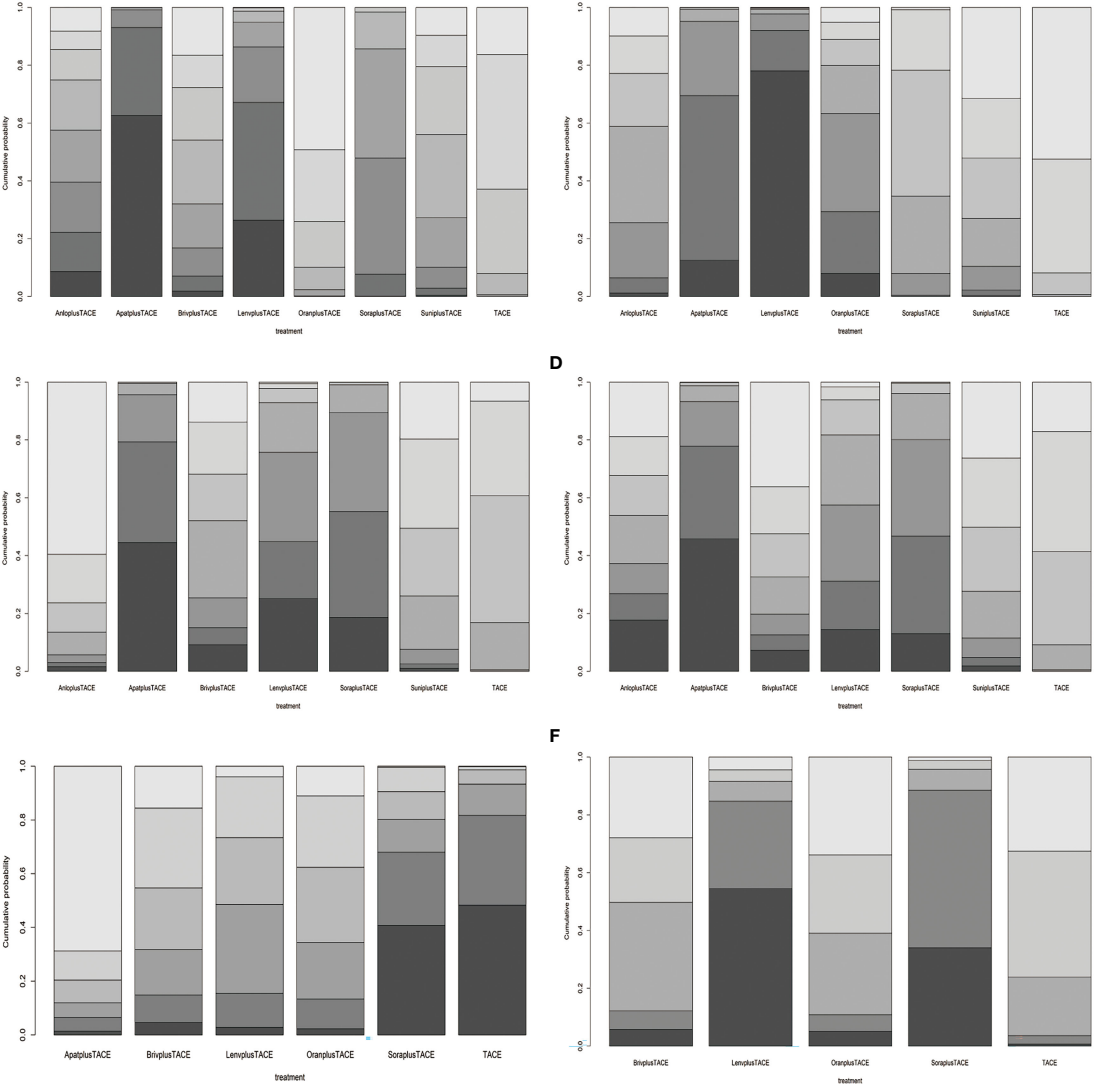
Relative rank plots based on probabilities of treatment strategies. **(A)** Overall survival. **(B)** Progression-free survival. **(C)** Objective response rate. **(D)** Disease control rate. **(E)** Adverse events. **(F)** ≥Grade-3 adverse events. Dark to light colors in the bar chart correspond to the ranking from top to bottom. LenvplusTACE, lenvatinib+TACE; BrivplusTACE, brivanib+TACE; ApatplusTACE, apatinib+TACE; AnloplusTACE, anlotinib+TACE; SuniplusTACE, sunitinib+TACE; SoraplusTACE, sorafenib+TACE; OranplusTACE, Orantinib+TACE.

For OS, 8 treatment strategies were documented altogether (**Figure 2**) and in comparison with TACE monotherapy and Oran+TACE, Apat+TACE, Lenv+TACE, and Sora+TACE demonstrated significant OS benefits (HR 0.62, 95% CI 0.50-0.75; HR 0.60, 95% CI 0.44-0.77; HR 0.68, 95% CI 0.49-0.88; HR 0.66, 95% CI 0.44-0.90; HR 0.78, 95% CI 0.68-0.86; HR 0.75, 95% CI 0.58-0.93) (**Figure 3**). Moreover, Apat+TACE supplied better OS than Sora+TACE and Suni+TACE (HR 0.80, 95% CI 0.67-0.95; HR 0.69, 95% CI 0.49-0.94). In the light of the ranking plot, Apat+TACE had the highest probability (62.2%) of delivering a better OS, followed by Lenv+TACE (40.6%), Sora+TACE (40.4%) and Anlo+TACE (18.3%) (**Figure 4**; **Supplementary Table 4**).

### 3.3 Progression-free survival

For PFS, 7 treatment strategies were documented altogether (**Figure 2**). Lenv+TACE and Apat+TACE were significantly ahead of Sora+TACE, Suni+TACE, and TACE monotherapy. Lenv+TACE offered better PFS than Sora+TACE (HR 0.53, 95%CI 0.32-0.88), Suni+TACE (HR 0.50, 95% CI 0.26-0.92), and TACE monotherapy (HR 0.46, 95% CI 0.28-0.73) (**Figure 3**). Similarly, Apat+TACE provided a better PFS than Sora+TACE (HR 0.69, 95% CI 0.53-0.93), Suni+TACE(HR 0.64, 95% CI 0.40-1.02), and TACE monotherapy(HR 0.60, 95% CI 0.46-0.75). In the light of the ranking plot, Lenv+TACE had the highest probability (78.9%) of providing a superior PFS, followed by Apat+TACE (58.1%), Oran+TACE (34.4%) and Anlo+TACE(32.7%) (**Figure 4**; **Supplementary Table 4**).

### 3.4 Objective response rate

For ORR, 7 treatment strategies were documented altogether (**Figure 2**). Compared with TACE monotherapy, Suni+TACE, and Anlo+TACE, Apat+TACE showed a significantly better ORR rate (OR 1.97, 95% CI 1.50-2.66; OR 2.05, 95% CI 1.01-4.04; OR 2.79 95% CI 1.00-8.32) (**Figure 3**). Sora+TACE was also demonstrated to have a significantly higher ORR rate than TACE monotherapy (OR 1.79, 95% CI 1.34-2.52). Additionally, no significant differences were found among the other treatments. In the light of the ranking plot, Apat+TACE had the highest probability of yielding a higher ORR rate (44.7%), followed by Sora+TACE (36.6%), Lenv+TACE (30.7%) and Briv+TACE (32.7%) (**Figure 4**; **Supplementary Table 4**).

### 3.5 Disease control rate

For DCR, 7 treatment strategies were documented altogether (**Figure 2**). Compared to the remaining six treatment measures, TACE monotherapy showed a lower DCR rate (OR 0.70, 95% CI 0.59-0.82; OR 0.75, 95% CI 0.63-0.88; OR 0.79, 95% CI 0.59-1.06; OR 0.88, 95% CI 0.49-1.57; OR 1.00, 95% CI 0.57-1.75; OR 0.98, 95% CI 0.69-1.45), but most differences were not statistically significant (**Figure 3**). Whereas there was no significant difference in the DCR of the six treatment strategies when compared with each other, Apat+TACE showed a higher DCR than Sora+TACE (OR 1.07, 95% CI 0.87-1.34), Lenv+TACE (OR 1.13, 95% CI 0.81-1.59), Anlo+TACE (OR 1.25, 95% CI 0.69-2.32), Briv+TACE (OR 1.42, 95% CI 0.80-2.58) and Suni+TACE(OR 1.40, 95% CI 0.96-2.17). In the light of the ranking plot, Apat+TACE had the highest probability of delivering a maximum DCR (45.6%), followed by Sora+TACE (33.7%), Lenv+TACE (26.1%) and Anlo+TACE (16.6%) (**Figure 4**; **Supplementary Table 4**).

### 3.6 ≥Grade-3 adverse events

**Figure 5:**
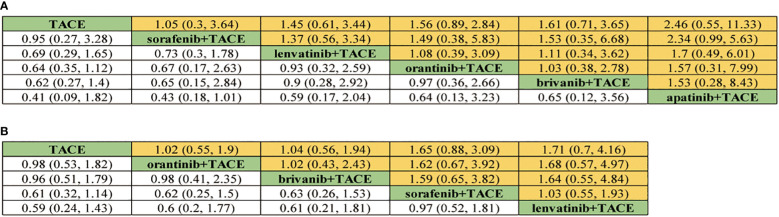
Pooled safety indicators estimates of network meta-analysis. **(A)** Pooled odds ratios (95% confidence intervals) of ≥Grade-3 adverse events. **(B)** Pooled odds ratios (95% confidence intervals) of adverse events.

For ≥G3-AEs, 6 treatment strategies were documented altogether (**Figure 2**). The various combined therapies did not significantly differ from one another (**Figure 5**). TACE demonstrated no statistically significant advantage in the low incidence of ≥G3-AEs while being a relatively safe treatment compared to other combined regimens (OR 0.95, 95% CI 0.27-3.28; OR 0.69, 95% CI 0.29-1.65; OR 0.64, 95% CI 0.35-1.12; OR 0.62, 95% CI 0.27-1.4; OR 0.41, 95% CI 0.09-1.82). In the light of the ranking plot, Apat+TACE was most likely to deliver the highest incidence of ≥G3-AEs (68.8%), followed by Briv+TACE (30.0%). Besides, TACE had the highest probability of delivering the safest treatment (48.2%), followed by Sora+TACE (27.3%) (**Figure 4**; **Supplementary Table 4**).

### 3.7 Adverse events

For AEs, 5 treatment strategies were documented altogether (**Figure 2**). There was no significant difference between the various treatment measures (**Figure 5**). Given the ranking plot, Oran+TACE had the highest probability of providing the safest treatment (34.3%), followed by TACE (44.2%). Besides, Sora+TACE was most likely to deliver the highest incidence of AEs (53.4%), followed by Lenv+TACE (53.4%) (**Figure 4**, **Supplementary Table 4**).

### 3.8 Publication bias and inconsistency analysis

**Figure 6:**
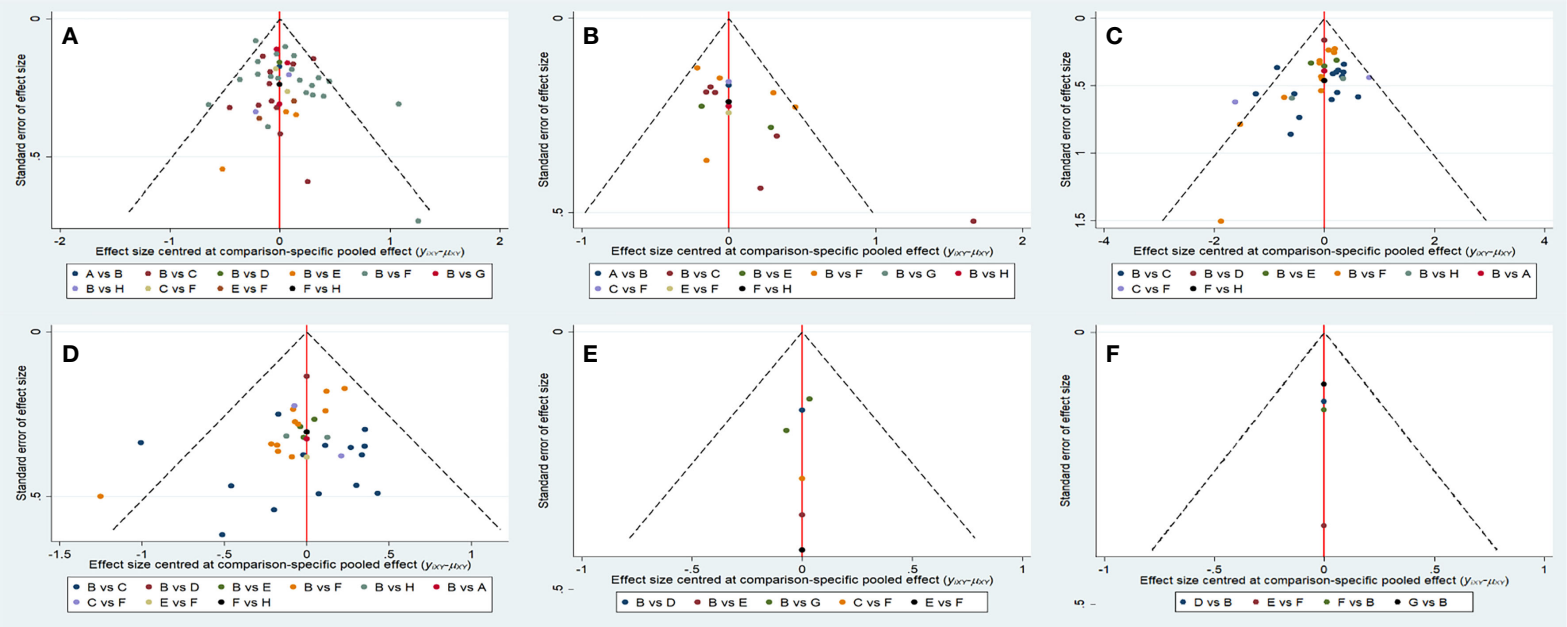
Funnel plots of each evaluation index. **(A)** Overall survival. **(B)** Progression-free survival. **(C)** Objective response rate. **(D)** Disease control rate. **(F)** ≥Grade-3 adverse events. **(E)** Adverse events. A, anlotinib+TACE; B, TACE; C, apatinib+TACE; D, brivanib+TACE; E, lenvatinib+TACE; F, sorafenib+TACE; G, orantinib+TACE; H, sunitinib+TACE.

The funnel plots of all indicators in the included study were nearly symmetrical, indicating no publication bias (**Figure 6**). Utilizing the node-splitting method, we found no inconsistency between direct and indirect comparison (**Supplementary Table 5**).

## 4 Discussion

So far, TACE combined with MTAs has become an essential approach for treating uHCC. Increasing combined therapies are applied in clinical practice. However, it is not easy to compare these therapies directly.

In this NMA, we primarily concentrated on comparing the efficacy and safety of TACE combined with MTAs. The results manifested that Apat+TACE had the best OS outcomes; Lenv+TACE and Sora+TACE placed second and third, respectively. The first three treatment strategies related to higher PFS outcomes were Lenv+TACE, Apat+TACE, and Oran+TACE. The first three treatment strategies related to higher ORR were Apat+TACE, Sora+TACE, and Lenv+TACE, while DCR was ranked similarly. In addition, there was no statistically significant difference among all treatment regimens with respect to the incidence of AEs and ≥G3-AEs. As a result, it can conclude that Apat+TACE was related to the best OS, ORR, and DCR, whereas Lenv+TACE was linked with the greatest PFS, with no extra AEs or ≥G3-AEs.

Apat, as an anti-angiogenic drug, preferentially inhibits VEGFR-2 tyrosine kinase, as well as slightly inhibits c-kit, c-src and, RET tyrosine kinases (56). It selectively binds to the intracellular ATP binding domain, inhibiting vascular endothelial cell proliferation and migration, reducing tumor angiogenesis, and inhibiting tumor formation (57). Furthermore, it can reverse the multidrug resistance caused by ABC protein and enhance the effectiveness of conventional anticancer drugs (58, 59). By inducing traditional chemotherapy medications and stimulating cell apoptosis, it can also have an anticancer effect (57). The above characteristics of apatinib are highly compatible with TACE treatment, which may be the important reason why Apat+TACE can stand out in many combined therapies.

For a long time, the comparison of survival time of HCC patients between Sora+TACE and Apat+TACE has been controversial. Qiu et al. (54) found that the PFS of the Apat+TACE group was shorter than that of the Sora+TACE group, while the OS of the two groups was not significantly different. Besides, Cao et al. (53) argued that in a retrospective study, the prognosis for both treatments was equivalent in patients with portal vein tumor thrombosis. It is worth mentioning that Apatinib has a tenfold higher affinity for VEGFR-2 tyrosine kinase than sorafenib (60). In our NMA, the OS and PFS of Apat+TACE were significantly better than those of Sora+TACE.

Regarding ORR, Apat+TACE had a higher ORR, suggesting a more significant proportion of patients with a 30% tumor decrease and maintenance for more than 4 weeks after Apat+TACE treatment. Because doctors or patients can clearly see the comparison of tumor bodies before and after therapy from the imaging, this sign is more intuitive for effect after treatment. However, ORR has a disadvantage in that it can only assess the efficacy of individual treatments and cannot reflect the overall advantages of the patient’s whole course of therapy. Apat+TACE fared better in terms of DCR, suggesting that it helped increase patient compliance and provided opportunities for more follow-up treatment in the future.

Hand-foot syndrome, hypertension, fatigue, and diarrhea were some of the most frequent adverse reactions to using TACE combined with MTAs. Compared to other combination therapy, Sora+TACE had more significant instances of erythema multiforme, rash, liver dysfunction, and alopecia. Thrombocytopenia and neutropenia were the side effects of Suni+TACE that occurred more frequently than those of other combination therapies. Pyrexia was the side effect that occurred more frequently with Oran+TACE than with other combination therapies. Liu et al. (43) reported in their study that two patients in the Apat+TACE group had to stop the trial for antihypertensive treatment due to severe hypertension but could continue the trial after treatment. Lu et al. (15) found that patients with severe hand-foot syndrome need to stop taking medication for two weeks and resume treatment after symptoms subside. Two other patients withdrew from treatment due to severe hand-foot syndrome. Lencioni et al. (13) reported that 4 deaths in the Sora+TACE group might be related to Sora. Zhu et al. (32) reported that patients with ≥G3-AEs in the Sora+TACE group needed to reduce the dosage of Sora or interrupt treatment. In their study, Kudo et al. (14) reported a case of death within 30 days after receiving Sora+TACE treatment. Chen et al. (36) reported in their study that 6 patients were forced to stop taking Lenv+TACE due to uncontrolled hypertension. However, in other studies, adverse reactions can be controlled by reducing drug dosage or providing symptomatic therapy without the occurrence of drug-related deaths. Therefore, it can be considered that combination therapy is within an acceptable range and is tolerable. In our NMA, there was no statistically significant difference in the incidence of AEs and ≥G3-AEs among various treatment strategies.

In our NMA, the OS, ORR, and DCR of Apat+TACE were better than that of Lenv+TACE apart from PFS, but there was no statistical difference between them. Similarly, Zhang et al. (61) compared the efficacy of TACE combined with four different tyrosine kinase inhibitors (TKIs) in their study. They deemed that the OS, PFS, ORR, and DCR of Lenv+TACE were better than those of Apat+TACE, but the difference was not statistically significant. We considered the reasons as follows: First, for OS and PFS indicators, since most of them are extracted from the Kaplan-Meier curve, different extractors had different subjective feelings, resulting in different extracted data. Second, we did not include those studies that had received systemic HCC treatment before the experiment or received other therapies at the same time during the experiment, which was different from the study of Zhang et al. (61). As a result, it is necessary to carry out a sizeable multi-center RCT to compare the efficacy between Apat+TACE and Lenv+TACE.

As an oral multikinase inhibitor, Sora directly suppresses tumor growth by regulating RAF/MEK/ERK pathway (62). It can also indirectly inhibit tumor growth and proliferation by inhibiting VEGFR and PDGFR to inhibit tumor neovascularization (63). There are already many meta-analyses comparing Sora+TACE with TACE. Zhang et al. (64) reported that Sora+TACE significantly outperformed TACE in terms of 1-year OS, 2 years OS, 3 years OS, 5 years OS, ORR, and DCR. Patients tolerated combination treatment well, despite the possibility of side effects related to Sora. Li et al. (65) mainly focused on the efficacy comparison between Sora+TACE and TACE. They thought that OS and time to progression (TTP) of combined treatment were significantly better than monotherapy. Chen et al. (66) believed that compared to the monotherapy group, the combined treatment group showed a significant increase in OS, TTP, and ORR. Also, there was no statistically significant difference in the incidence of AEs between the two treatment groups.

In our NMA, the Apat+TACE group had significantly better OS, PFS, ORR, and DCR than the TACE group, while there was no statistically significant difference in AEs and ≥G3-AEs between the two groups. Many meta-analyses currently exist comparing Apat+TACE with TACE. Wei et al. (67) indicated that compared with the TACE group, the Apat+TACE group had significant benefits in 6 months OS, 1 year OS, and 2 years OS. Exception for the incidence of hand-foot syndrome, proteinuria, hypertension, and diarrhea, the Apat+TACE group was significantly higher than the TACE group. There was no statistical difference in the incidence of other adverse reactions. At the same time, Gong et al. (68) also concluded similarly to the foregoing.

Lenv, as an oral multi-kinase inhibitor, inhibits VEGFR-1/2/3, fibroblast growth factor receptor (FGFR) 1-4, and PDGFR-α, RET, and KIT targets, thereby inhibiting tumor cell growth and tumor angiogenesis (69). Liu et al. (70) conducted a meta-analysis comparing Lenv+TACE with Sora+TACE and found that the OS and PFS of the Lenv+TACE group were significantly better than those of Sora+TACE. In terms of safety, the incidence of hypertension and proteinuria was significantly higher in the Lenv+TACE group than in the Sora+TACE group, while the opposite was true for the hand-foot syndrome. The PFS of the Lenv+TACE group was significantly better than that of the Sora+TACE group in our NMA. In terms of OS, AEs, and ≥G3-AEs, there was no statistically significant difference between the two groups. As a result, there is disagreement on the efficacy of the comparison between Lenv+TACE and Sora+TACE, and a large, multicenter RCT is required to validate it.

Suni, as a tyrosine kinase inhibitor (TKI), targets PDGF-α/β, VEGFR-1/2/3, KIT, FLT-3, CSF-1, and RET. Briv, as a TKI, selectively inhibits VEGFR and FGFR. Anlo, as a TKI, targets VEGFR, FGFR, PDGFR, and c-kit (71). Oran, as a TKI, targets VEGFR-2 and PDGFR-β. Through the above mechanisms, it exerts its anti-tumor proliferation and anti-tumor angiogenesis effects (72). Due to the limited number of studies on the combination of the three MTAs combined with TACE for the treatment of HCC, there is no meta-analysis on the combination of the MTAs and TACE for the treatment of HCC.

Our NMA had the following advantages. Firstly, studies that used other systemic therapies before or during MTAs+TACE therapy or TACE monotherapy were excluded to reflect the efficacy of MTAs+TACE more accurately. Secondly, it summarized the current research on MTAs combined with TACE to compare the efficacy and safety of various MTAs+TACE.

At the same time, however, there were also some limitations in the NMA. Firstly, among the 45 studies we included, only 5 were related to the comparison between MTAs and TACE, while the rest was compared between MTAs+TACE and TACE. Therefore, merging and comparing the comparisons of MTAs+TACE may undermine the credibility of the research. Secondly, since the HR and 95% CIs of the OS and PFS were rarely directly provided by the original study, we needed to extract data from the curve. Due to the subjective nature of extracting data, the accuracy of HR and its CIs related to OS and PFS may be affected. Thirdly, out of the 45 studies we included, only 10 were RCTs, which may bring confounding factors to our research and lead to the risk of selective bias.

The network meta-analysis showed that apatinib+TACE displayed the best OS, ORR, and DCR with no additional AEs and ≥G3-AEs. Therefore, for the treatment scheme of MTAs combined with TACE, apatinib+TACE may be the most effective treatment.

## Data availability statement

The original contributions presented in the study are included in the article/**Supplementary Material**. Further inquiries can be directed to the corresponding author.

## Author contributions

JL was responsible for writing the manuscript. BC and ZL analyzed the data. BC was responsible for research and design. JL, BC, and ZL searched the literature together. BC participated in the revision of the manuscript. All authors contributed to the article and approved the submitted version.
