# Supplementary material for: Comparative efficacy and safety of molecular targeted agents combined with transarterial chemoembolization in the treatment of unresectable hepatocellular carcinoma: a network meta-analysis
Source: Front Oncol. 2023 May 17;13:1179431. doi: 10.3389/fonc.2023.1179431 (PMC10230082; doi:10.3389/fonc.2023.1179431)
Supplement: Supplementary file 1 [file Table_1.docx]

Supplementary Material

Comparative efficacy and safety of molecular targeted agents combined with transarterial chemoembolization in the treatment of unresectable hepatocellular carcinoma: a network meta-analysis

Jiaye Long^1^, Baoxiang Chen^1*^, Zhaohui Liu^2^

^1^Department of Interventional Radiology, Inner Mongolia Forestry General Hospital, The Second Clinical Medical School of Inner Mongolia University for The Nationalities, Yakeshi, Inner Mongolia, China

^2^ Department of Urology, Inner Mongolia Forestry General Hospital, The Second Clinical Medical School of Inner Mongolia University for The Nationalities, Yakeshi, Inner Mongolia, China

*** Correspondence:** Baoxiang Chen: 280496340@qq.com

**
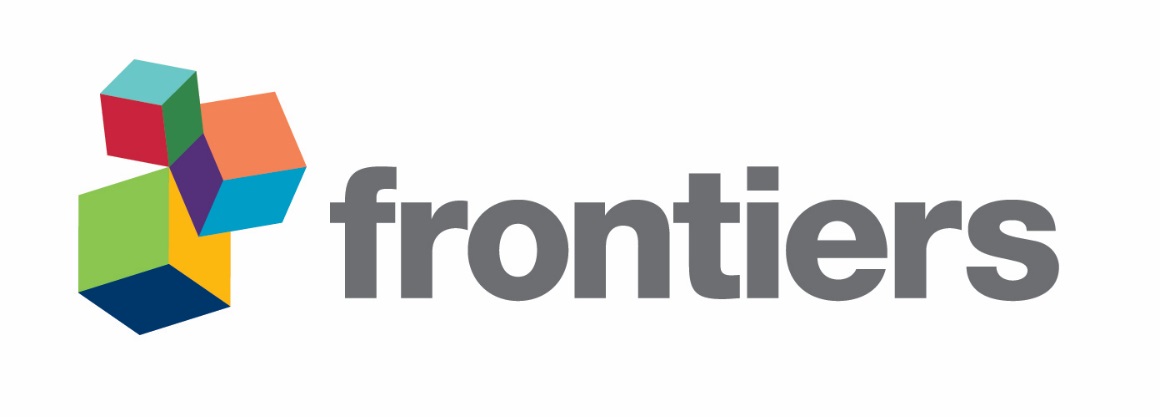
**

**Supplementary Table 1. Results of full search strategy of database.**

| **Search strategy in Pubmed** | | |
| --- | --- | --- |
| Search | Query | Results |
| #1 | ("Liver Neoplasms"[Mesh]) OR (((((((((((((((((((((Neoplasms, Hepatic[Title/Abstract]) OR (Neoplasms, Liver[Title/Abstract])) OR (Liver Neoplasm[Title/Abstract])) OR (Neoplasm, Liver[Title/Abstract])) OR (Hepatic Neoplasms[Title/Abstract])) OR (Hepatic Neoplasm[Title/Abstract])) OR (Neoplasm, Hepatic[Title/Abstract])) OR (Cancer of Liver[Title/Abstract])) OR (Hepatocellular Cancer[Title/Abstract])) OR (Cancers, Hepatocellular[Title/Abstract])) OR (Hepatocellular Cancers[Title/Abstract])) OR (Hepatic Cancer[Title/Abstract])) OR (Cancer, Hepatic[Title/Abstract])) OR (Cancers, Hepatic[Title/Abstract])) OR (Hepatic Cancers[Title/Abstract])) OR (Liver Cancer[Title/Abstract])) OR (Cancer, Liver[Title/Abstract])) OR (Cancers, Liver[Title/Abstract])) OR (Liver Cancers[Title/Abstract])) OR (Cancer of the Liver[Title/Abstract])) OR (Cancer, Hepatocellular[Title/Abstract])) | 204,316 |
| #2 | ("Sorafenib"[Mesh]) OR (((((((((((((((Nexavar[Title/Abstract]) OR (BAY 43-9006[Title/Abstract])) OR (BAY 43 9006[Title/Abstract])) OR (BAY 439006[Title/Abstract])) OR (Sorafenib N-Oxide[Title/Abstract])) OR (Sorafenib N Oxide[Title/Abstract])) OR (BAY-673472[Title/Abstract])) OR (BAY 673472[Title/Abstract])) OR (BAY 545-9085[Title/Abstract])) OR (BAY 545 9085[Title/Abstract])) OR (BAY 5459085[Title/Abstract])) OR (BAY-545-9085[Title/Abstract])) OR (BAY5459085[Title/Abstract])) OR (Sorafenib Tosylate[Title/Abstract])) OR (4-(4-(3-(4-Chloro-3-trifluoromethylphenyl)ureido)phenoxy)pyridine-2-carboxylic acid methyamide-4-methylbenzenesulfonate[Title/Abstract])) | 6,247 |
| #3 | (("Liver Neoplasms"[Mesh]) OR (((((((((((((((((((((Neoplasms, Hepatic[Title/Abstract]) OR (Neoplasms, Liver[Title/Abstract])) OR (Liver Neoplasm[Title/Abstract])) OR (Neoplasm, Liver[Title/Abstract])) OR (Hepatic Neoplasms[Title/Abstract])) OR (Hepatic Neoplasm[Title/Abstract])) OR (Neoplasm, Hepatic[Title/Abstract])) OR (Cancer of Liver[Title/Abstract])) OR (Hepatocellular Cancer[Title/Abstract])) OR (Cancers, Hepatocellular[Title/Abstract])) OR (Hepatocellular Cancers[Title/Abstract])) OR (Hepatic Cancer[Title/Abstract])) OR (Cancer, Hepatic[Title/Abstract])) OR (Cancers, Hepatic[Title/Abstract])) OR (Hepatic Cancers[Title/Abstract])) OR (Liver Cancer[Title/Abstract])) OR (Cancer, Liver[Title/Abstract])) OR (Cancers, Liver[Title/Abstract])) OR (Liver Cancers[Title/Abstract])) OR (Cancer of the Liver[Title/Abstract])) OR (Cancer, Hepatocellular[Title/Abstract]))) AND (("Sorafenib"[Mesh]) OR (((((((((((((((Nexavar[Title/Abstract]) OR (BAY 43-9006[Title/Abstract])) OR (BAY 43 9006[Title/Abstract])) OR (BAY 439006[Title/Abstract])) OR (Sorafenib N-Oxide[Title/Abstract])) OR (Sorafenib N Oxide[Title/Abstract])) OR (BAY-673472[Title/Abstract])) OR (BAY 673472[Title/Abstract])) OR (BAY 545-9085[Title/Abstract])) OR (BAY 545 9085[Title/Abstract])) OR (BAY 5459085[Title/Abstract])) OR (BAY-545-9085[Title/Abstract])) OR (BAY5459085[Title/Abstract])) OR (Sorafenib Tosylate[Title/Abstract])) OR (4-(4-(3-(4-Chloro-3-trifluoromethylphenyl)ureido)phenoxy)pyridine-2-carboxylic acid methyamide-4-methylbenzenesulfonate[Title/Abstract]))) | 3,068 |
| #4 | ("Sunitinib"[Mesh]) OR (((((((((5-(5-Fluoro-2-oxo-1,2-dihydroindolylidenemethyl)-2,4-dimethyl-1H-pyrrole-3-carboxylic acid (2-diethylaminoethyl)amide[Title/Abstract]) OR (Sunitinib Malate[Title/Abstract])) OR (Sutent[Title/Abstract])) OR (SU 11248[Title/Abstract])) OR (SU011248[Title/Abstract])) OR (SU 011248[Title/Abstract])) OR (SU-011248[Title/Abstract])) OR (SU11248[Title/Abstract])) OR (SU-11248[Title/Abstract])) | 4,300 |
| #5 | (("Liver Neoplasms"[Mesh]) OR (((((((((((((((((((((Neoplasms, Hepatic[Title/Abstract]) OR (Neoplasms, Liver[Title/Abstract])) OR (Liver Neoplasm[Title/Abstract])) OR (Neoplasm, Liver[Title/Abstract])) OR (Hepatic Neoplasms[Title/Abstract])) OR (Hepatic Neoplasm[Title/Abstract])) OR (Neoplasm, Hepatic[Title/Abstract])) OR (Cancer of Liver[Title/Abstract])) OR (Hepatocellular Cancer[Title/Abstract])) OR (Cancers, Hepatocellular[Title/Abstract])) OR (Hepatocellular Cancers[Title/Abstract])) OR (Hepatic Cancer[Title/Abstract])) OR (Cancer, Hepatic[Title/Abstract])) OR (Cancers, Hepatic[Title/Abstract])) OR (Hepatic Cancers[Title/Abstract])) OR (Liver Cancer[Title/Abstract])) OR (Cancer, Liver[Title/Abstract])) OR (Cancers, Liver[Title/Abstract])) OR (Liver Cancers[Title/Abstract])) OR (Cancer of the Liver[Title/Abstract])) OR (Cancer, Hepatocellular[Title/Abstract]))) AND (("Sunitinib"[Mesh]) OR (((((((((5-(5-Fluoro-2-oxo-1,2-dihydroindolylidenemethyl)-2,4-dimethyl-1H-pyrrole-3-carboxylic acid (2-diethylaminoethyl)amide[Title/Abstract]) OR (Sunitinib Malate[Title/Abstract])) OR (Sutent[Title/Abstract])) OR (SU 11248[Title/Abstract])) OR (SU011248[Title/Abstract])) OR (SU 011248[Title/Abstract])) OR (SU-011248[Title/Abstract])) OR (SU11248[Title/Abstract])) OR (SU-11248[Title/Abstract]))) | 195 |
| #6 | ("brivanib" [Supplementary Concept]) OR (((((((BMS 540215[Title/Abstract]) OR (BMS540215[Title/Abstract])) OR (BMS-540215[Title/Abstract])) OR (BMS 582664[Title/Abstract])) OR (BMS582664[Title/Abstract])) OR (BMS-582664[Title/Abstract])) OR (brivanib alaninate[Title/Abstract])) | 76 |
| #7 | (("Liver Neoplasms"[Mesh]) OR (((((((((((((((((((((Neoplasms, Hepatic[Title/Abstract]) OR (Neoplasms, Liver[Title/Abstract])) OR (Liver Neoplasm[Title/Abstract])) OR (Neoplasm, Liver[Title/Abstract])) OR (Hepatic Neoplasms[Title/Abstract])) OR (Hepatic Neoplasm[Title/Abstract])) OR (Neoplasm, Hepatic[Title/Abstract])) OR (Cancer of Liver[Title/Abstract])) OR (Hepatocellular Cancer[Title/Abstract])) OR (Cancers, Hepatocellular[Title/Abstract])) OR (Hepatocellular Cancers[Title/Abstract])) OR (Hepatic Cancer[Title/Abstract])) OR (Cancer, Hepatic[Title/Abstract])) OR (Cancers, Hepatic[Title/Abstract])) OR (Hepatic Cancers[Title/Abstract])) OR (Liver Cancer[Title/Abstract])) OR (Cancer, Liver[Title/Abstract])) OR (Cancers, Liver[Title/Abstract])) OR (Liver Cancers[Title/Abstract])) OR (Cancer of the Liver[Title/Abstract])) OR (Cancer, Hepatocellular[Title/Abstract]))) AND (("brivanib" [Supplementary Concept]) OR (((((((BMS 540215[Title/Abstract]) OR (BMS540215[Title/Abstract])) OR (BMS-540215[Title/Abstract])) OR (BMS 582664[Title/Abstract])) OR (BMS582664[Title/Abstract])) OR (BMS-582664[Title/Abstract])) OR (brivanib alaninate[Title/Abstract]))) | 22 |
| #8 | ("anlotinib" [Supplementary Concept]) OR (AL3818[Title/Abstract]) | 248 |
| #9 | (("Liver Neoplasms"[Mesh]) OR (((((((((((((((((((((Neoplasms, Hepatic[Title/Abstract]) OR (Neoplasms, Liver[Title/Abstract])) OR (Liver Neoplasm[Title/Abstract])) OR (Neoplasm, Liver[Title/Abstract])) OR (Hepatic Neoplasms[Title/Abstract])) OR (Hepatic Neoplasm[Title/Abstract])) OR (Neoplasm, Hepatic[Title/Abstract])) OR (Cancer of Liver[Title/Abstract])) OR (Hepatocellular Cancer[Title/Abstract])) OR (Cancers, Hepatocellular[Title/Abstract])) OR (Hepatocellular Cancers[Title/Abstract])) OR (Hepatic Cancer[Title/Abstract])) OR (Cancer, Hepatic[Title/Abstract])) OR (Cancers, Hepatic[Title/Abstract])) OR (Hepatic Cancers[Title/Abstract])) OR (Liver Cancer[Title/Abstract])) OR (Cancer, Liver[Title/Abstract])) OR (Cancers, Liver[Title/Abstract])) OR (Liver Cancers[Title/Abstract])) OR (Cancer of the Liver[Title/Abstract])) OR (Cancer, Hepatocellular[Title/Abstract]))) AND (("anlotinib" [Supplementary Concept]) OR (AL3818[Title/Abstract])) | 16 |
| #10 | ("apatinib" [Supplementary Concept]) OR (((((rivoceranib mesylate[Title/Abstract]) OR (YN968D1[Title/Abstract])) OR (YN-968D1[Title/Abstract])) OR (rivoceranib[Title/Abstract])) OR (apatinib mesylate[Title/Abstract])) | 578 |
| #11 | (("Liver Neoplasms"[Mesh]) OR (((((((((((((((((((((Neoplasms, Hepatic[Title/Abstract]) OR (Neoplasms, Liver[Title/Abstract])) OR (Liver Neoplasm[Title/Abstract])) OR (Neoplasm, Liver[Title/Abstract])) OR (Hepatic Neoplasms[Title/Abstract])) OR (Hepatic Neoplasm[Title/Abstract])) OR (Neoplasm, Hepatic[Title/Abstract])) OR (Cancer of Liver[Title/Abstract])) OR (Hepatocellular Cancer[Title/Abstract])) OR (Cancers, Hepatocellular[Title/Abstract])) OR (Hepatocellular Cancers[Title/Abstract])) OR (Hepatic Cancer[Title/Abstract])) OR (Cancer, Hepatic[Title/Abstract])) OR (Cancers, Hepatic[Title/Abstract])) OR (Hepatic Cancers[Title/Abstract])) OR (Liver Cancer[Title/Abstract])) OR (Cancer, Liver[Title/Abstract])) OR (Cancers, Liver[Title/Abstract])) OR (Liver Cancers[Title/Abstract])) OR (Cancer of the Liver[Title/Abstract])) OR (Cancer, Hepatocellular[Title/Abstract]))) AND (("apatinib" [Supplementary Concept]) OR (((((rivoceranib mesylate[Title/Abstract]) OR (YN968D1[Title/Abstract])) OR (YN-968D1[Title/Abstract])) OR (rivoceranib[Title/Abstract])) OR (apatinib mesylate[Title/Abstract]))) | 89 |
| #12 | ("orantinib" [Supplementary Concept]) OR (((((((((5-((1,2-dihydro-2-oxo-3H-indol-3-ylidene)methyl)-2,4-dimethyl-1H-pyrrole-3-propanoic acid[Title/Abstract]) OR ((Z)-3-(2,4-dimethyl-5-(2-oxo-1,2-dihydro-indol-3-ylidenemethyl)-1H-pyrrol-3-yl)-propionic acid[Title/Abstract])) OR ((Z)-3-(2,4-dimethyl-5-(2-oxo-1,2-dihydro-indol-3-ylidenemethyl)-1H-pyrrol-3-yl)propionic acid[Title/Abstract])) OR (SU 6668[Title/Abstract])) OR (TSU-68[Title/Abstract])) OR (SU006668[Title/Abstract])) OR (TSU 68[Title/Abstract])) OR (SU-6668[Title/Abstract])) OR (SU6668[Title/Abstract])) | 137 |
| #13 | (("Liver Neoplasms"[Mesh]) OR (((((((((((((((((((((Neoplasms, Hepatic[Title/Abstract]) OR (Neoplasms, Liver[Title/Abstract])) OR (Liver Neoplasm[Title/Abstract])) OR (Neoplasm, Liver[Title/Abstract])) OR (Hepatic Neoplasms[Title/Abstract])) OR (Hepatic Neoplasm[Title/Abstract])) OR (Neoplasm, Hepatic[Title/Abstract])) OR (Cancer of Liver[Title/Abstract])) OR (Hepatocellular Cancer[Title/Abstract])) OR (Cancers, Hepatocellular[Title/Abstract])) OR (Hepatocellular Cancers[Title/Abstract])) OR (Hepatic Cancer[Title/Abstract])) OR (Cancer, Hepatic[Title/Abstract])) OR (Cancers, Hepatic[Title/Abstract])) OR (Hepatic Cancers[Title/Abstract])) OR (Liver Cancer[Title/Abstract])) OR (Cancer, Liver[Title/Abstract])) OR (Cancers, Liver[Title/Abstract])) OR (Liver Cancers[Title/Abstract])) OR (Cancer of the Liver[Title/Abstract])) OR (Cancer, Hepatocellular[Title/Abstract]))) AND (("orantinib" [Supplementary Concept]) OR (((((((((5-((1,2-dihydro-2-oxo-3H-indol-3-ylidene)methyl)-2,4-dimethyl-1H-pyrrole-3-propanoic acid[Title/Abstract]) OR ((Z)-3-(2,4-dimethyl-5-(2-oxo-1,2-dihydro-indol-3-ylidenemethyl)-1H-pyrrol-3-yl)-propionic acid[Title/Abstract])) OR ((Z)-3-(2,4-dimethyl-5-(2-oxo-1,2-dihydro-indol-3-ylidenemethyl)-1H-pyrrol-3-yl)propionic acid[Title/Abstract])) OR (SU 6668[Title/Abstract])) OR (TSU-68[Title/Abstract])) OR (SU006668[Title/Abstract])) OR (TSU 68[Title/Abstract])) OR (SU-6668[Title/Abstract])) OR (SU6668[Title/Abstract]))) | 16 |
| #14 | ("lenvatinib" [Supplementary Concept]) OR ((((((((((((((((((4-(3-chloro-4-(N'-cyclopropylureido)phenoxy)-7-methoxyquinoline-6-carboxamide[Title/Abstract]) OR (4-(3-chloro-4-((cyclopropylaminocarbonyl)amino)phenoxy)-7-methoxy-6-quinolinecarbox[Title/Abstract])) OR (amide[Title/Abstract])) OR (Lenvima[Title/Abstract])) OR (E 7080[Title/Abstract])) OR (E-7080[Title/Abstract])) OR (ER-203492-00[Title/Abstract])) OR (E7080[Title/Abstract])) OR (E-7080 mesylate[Title/Abstract])) OR (E7080 mesylate[Title/Abstract])) OR (lenvatinib metabolite M2[Title/Abstract])) OR (4-(3-chloro-4-(((cyclopropylamino)carbonyl)amino)phenoxy)-7-hydroxy-6-quinolinecarbo[Title/Abstract])) OR (xamide[Title/Abstract])) OR (lenvatinib mesylate[Title/Abstract])) OR (lenvatinib methanesulfonate[Title/Abstract])) OR (N-(4-((6-carbamoyl-7-methoxyquinolin-4-yl)oxy)-2-chlorophenyl)-N'-cyclopropylurea m[Title/Abstract])) OR (onomethanesulfonate[Title/Abstract])) OR (lenvatinib mesilate[Title/Abstract])) | 47,897 |
| #15 | (("Liver Neoplasms"[Mesh]) OR (((((((((((((((((((((Neoplasms, Hepatic[Title/Abstract]) OR (Neoplasms, Liver[Title/Abstract])) OR (Liver Neoplasm[Title/Abstract])) OR (Neoplasm, Liver[Title/Abstract])) OR (Hepatic Neoplasms[Title/Abstract])) OR (Hepatic Neoplasm[Title/Abstract])) OR (Neoplasm, Hepatic[Title/Abstract])) OR (Cancer of Liver[Title/Abstract])) OR (Hepatocellular Cancer[Title/Abstract])) OR (Cancers, Hepatocellular[Title/Abstract])) OR (Hepatocellular Cancers[Title/Abstract])) OR (Hepatic Cancer[Title/Abstract])) OR (Cancer, Hepatic[Title/Abstract])) OR (Cancers, Hepatic[Title/Abstract])) OR (Hepatic Cancers[Title/Abstract])) OR (Liver Cancer[Title/Abstract])) OR (Cancer, Liver[Title/Abstract])) OR (Cancers, Liver[Title/Abstract])) OR (Liver Cancers[Title/Abstract])) OR (Cancer of the Liver[Title/Abstract])) OR (Cancer, Hepatocellular[Title/Abstract]))) AND (("lenvatinib" [Supplementary Concept]) OR ((((((((((((((((((4-(3-chloro-4-(N'-cyclopropylureido)phenoxy)-7-methoxyquinoline-6-carboxamide[Title/Abstract]) OR (4-(3-chloro-4-((cyclopropylaminocarbonyl)amino)phenoxy)-7-methoxy-6-quinolinecarbox[Title/Abstract])) OR (amide[Title/Abstract])) OR (Lenvima[Title/Abstract])) OR (E 7080[Title/Abstract])) OR (E-7080[Title/Abstract])) OR (ER-203492-00[Title/Abstract])) OR (E7080[Title/Abstract])) OR (E-7080 mesylate[Title/Abstract])) OR (E7080 mesylate[Title/Abstract])) OR (lenvatinib metabolite M2[Title/Abstract])) OR (4-(3-chloro-4-(((cyclopropylamino)carbonyl)amino)phenoxy)-7-hydroxy-6-quinolinecarbo[Title/Abstract])) OR (xamide[Title/Abstract])) OR (lenvatinib mesylate[Title/Abstract])) OR (lenvatinib methanesulfonate[Title/Abstract])) OR (N-(4-((6-carbamoyl-7-methoxyquinolin-4-yl)oxy)-2-chlorophenyl)-N'-cyclopropylurea m[Title/Abstract])) OR (onomethanesulfonate[Title/Abstract])) OR (lenvatinib mesilate[Title/Abstract]))) | 446 |
|  | **Search strategy in Webofscience** |  |
| Search | Query | Results |
| 1 | TS=( Liver Neoplasms OR Neoplasms, Hepatic OR Neoplasms, Liver OR Liver Neoplasm OR Neoplasm, Liver OR Hepatic Neoplasms OR Hepatic Neoplasm OR Neoplasm, Hepatic OR Cancer of Liver OR Hepatocellular Cancer OR Cancers, Hepatocellular OR Hepatocellular Cancers OR Hepatic Cancer OR Cancer, Hepatic OR Cancers, Hepatic OR Hepatic Cancers OR Liver Cancer OR Cancer, Liver OR Cancers, Liver OR Liver Cancers OR Cancer of the Liver OR Cancer, Hepatocellular) | 208,324 |
| 2 | TS=(Chemoembolization, Therapeutic OR Therapeutic Chemoembolization OR Chemoembolizations, Therapeutic OR Therapeutic Chemoembolizations) | 2,222 |
| 3 | TS=(sorafenib OR Nexavar OR BAY 43-9006 OR BAY 43 9006 OR BAY 439006 OR Sorafenib N-Oxide OR Sorafenib N Oxide OR BAY-673472 OR BAY 673472 OR BAY 545-9085 OR BAY 545 9085 OR BAY 5459085 OR BAY-545-9085 OR BAY5459085 OR Sorafenib Tosylate OR 4-(4-(3-(4-Chloro-3-trifluoromethylphenyl)ureido)phenoxy)pyridine-2-carboxylic acid methyamide-4-methylbenzenesulfonate) | 18,988 |
| 4 | #1 AND #2 AND #3 | 252 |
| 5 | TS=( Sunitinib OR 5-(5-Fluoro-2-oxo-1,2-dihydroindolylidenemethyl)-2,4-dimethyl-1H-pyrrole-3-carboxylic acid (2-diethylaminoethyl)amide OR Sunitinib Malate OR Sutent ORSU 11248 OR SU011248 OR SU 011248 OR SU-011248 OR SU11248 OR SU-11248) | 12,296 |
| 6 | #1 AND #2 AND #5 | 11 |
| 7 | TS=(Brivanib OR BMS 540215 OR BMS540215 OR BMS-540215 OR BMS 582664 OR BMS582664 OR BMS-582664 OR brivanib alaninate) | 293 |
| 8 | #1 AND #2 AND #7 | 7 |
| 9 | TS=( Anlotinib OR AL3818) | 886 |
| 10 | #1 AND #2 AND #9 | 1 |
| 11 | TS=(Apatinib OR rivoceranib mesylate OR YN968D1 OR YN-968D1 OR rivoceranib OR apatinib mesylate) | 1,554 |
| 12 | #1 AND #2 AND #11 | 9 |
| 13 | TS=(Orantinib OR 5-((1,2-dihydro-2-oxo-3H-indol-3-ylidene)methyl)-2,4-dimethyl-1H-pyrrole-3-propanoic acid OR (Z)-3-(2,4-dimethyl-5-(2-oxo-1,2-dihydro-indol-3-ylidenemethyl)-1H-pyrrol-3-yl)-propionic acid OR (Z)-3-(2,4-dimethyl-5-(2-oxo-1,2-dihydro-indol-3-ylidenemethyl)-1H-pyrrol-3-yl)propionic acid OR SU 6668 OR TSU-68 OR SU006668 OR TSU 68 OR SU-6668 OR SU6668) | 169 |
| 14 | #1 AND # 2 AND #13 | 0 |
| 15 | TS=(lenvatinib OR 4-(3-chloro-4-(N'-cyclopropylureido)phenoxy)-7-methoxyquinoline-6-carboxamide OR 4-(3-chloro-4-((cyclopropylaminocarbonyl)amino)phenoxy)-7-methoxy-6-quinolinecarboxamide OR Lenvima OR E 7080 OR E-7080 OR ER-203492-00 OR E7080 OR E-7080 mesylate OR E7080 mesylate OR E7080 mesylate OR lenvatinib metabolite M2 OR 4-(3-chloro-4-(((cyclopropylamino)carbonyl)amino)phenoxy)-7-hydroxy-6-quinolinecarboxamide OR lenvatinib mesylate OR lenvatinib methanesulfonate OR N-(4-((6-carbamoyl-7-methoxyquinolin-4-yl)oxy)-2-chlorophenyl)-N'-cyclopropylurea monomethanesulfonate OR lenvatinib mesilate) | 2,699 |
| 16 | #1 AND # 2 AND #15 | 40 |
|  | **Search strategy in Embase** |  |
| No | Query | Results |
| #1 | 'liver tumor'/exp OR 'liver tumor' | 354,978 |
| #2 | 'neoplasms, hepatic':ti,ab OR 'neoplasms, liver':ti,ab OR 'liver neoplasm':ti,ab OR 'neoplasm, liver':ti,ab OR 'hepatic neoplasms':ti,ab OR 'hepatic neoplasms':ti,ab OR 'hepatic neoplasm':ti,ab OR 'neoplasm, hepatic':ti,ab OR 'cancer of liver':ti,ab OR 'hepatocellular cancer':ti,ab OR 'cancers, hepatocellular':ti,ab OR 'hepatocellular cancers':ti,ab OR 'hepatic cancer':ti,ab OR 'cancer, hepatic':ti,ab OR 'cancers, hepatic':ti,ab OR 'hepatic cancers':ti,ab OR 'liver cancer':ti,ab OR 'cancer, liver':ti,ab OR 'cancers, liver':ti,ab OR 'liver cancers':ti,ab OR 'cancer of the liver':ti,ab OR 'cancer, hepatocellular':ti,ab | 49,875 |
| #3 | #1 OR #2 | 363,352 |
| #4 | 'chemoembolization'/exp OR 'chemoembolization' | 23,747 |
| #5 | 'therapeutic chemoembolization':ti,ab OR 'chemoembolizations, therapeutic':ti,ab OR 'therapeutic chemoembolizations':ti,ab | 1 |
| #6 | #4 OR #5 | 23,747 |
| #7 | 'sorafenib'/exp OR 'sorafenib' | 37,606 |
| #8 | 'nexavar':ti,ab OR 'bay 43-9006':ti,ab OR 'bay 43 9006':ti,ab OR 'bay 439006':ti,ab OR 'sorafenib n-oxide':ti,ab OR 'sorafenib n oxide':ti,ab OR 'bay-673472':ti,ab OR 'bay 673472':ti,ab OR 'bay 545-9085':ti,ab OR 'or bay 545 9085':ti,ab OR 'bay 5459085':ti,ab OR 'bay-545-9085':ti,ab OR 'bay5459085':ti,ab OR 'sorafenib tosylate':ti,ab OR '4-(4-(3-(4-chloro-3-trifluoromethylphenyl)ureido)phenoxy)pyridine-2-carboxylic acid methyamide-4-methylbenzenesulfonate':ti,ab | 579 |
| #9 | #7 OR #8 | 37,618 |
| #10 | #3 AND #6 AND #9 | 3,593 |
| #11 | 'sunitinib'/exp OR 'sunitinib' | 27,7611 |
| #12 | '5-(5-fluoro-2-oxo-1,2-dihydroindolylidenemethyl)-2,4-dimethyl-1h-pyrrole-3-carboxylic acid (2-diethylaminoethyl)amide':ti,ab OR 'sunitinib malate':ti,ab OR 'sutent orsu 11248':ti,ab OR 'su011248':ti,ab OR 'su 011248':ti,ab OR 'su-011248':ti,ab OR 'su11248':ti,ab OR 'su-11248':ti,ab | 654 |
| #13 | #11 OR #12 | 27,623 |
| #14 | #3 AND #6 AND #13 | 426 |
| #15 | 'brivanib'/exp OR 'brivanib' | 1,030 |
| #16 | 'bms 540215':ti,ab OR 'bms540215':ti,ab OR 'bms-540215':ti,ab OR 'bms 582664':ti,ab OR 'bms582664':ti,ab OR 'bms-582664':ti,ab OR 'brivanib alaninate':ti,ab | 71 |
| #17 | #15 OR #16 | 1,031 |
| #18 | #3 AND #6 AND #17 | 200 |
| #19 | 'catequentinib'/exp OR 'catequentinib' | 1,359 |
| #20 | 'anlotinib':ti,ab OR 'al3818':ti,ab | 985 |
| #21 | #19 OR #20 | 1,411 |
| #22 | #3 AND #6 AND #21 | 31 |
| #23 | 'rivoceranib'/exp OR 'rivoceranib' | 2,462 |
| #24 | 'apatinib':ti,ab OR 'rivoceranib mesylate':ti,ab OR 'yn968d1':ti,ab OR 'yn-968d1':ti,ab OR 'rivoceranib':ti,ab OR 'apatinib mesylate':ti,ab | 1,768 |
| #25 | #23 OR #24 | 2,590 |
| #26 | #3 AND #6 AND #25 | 183 |
| #27 | 'orantinib'/exp OR 'orantinib' | 1,032 |
| #28 | '5-((1,2-dihydro-2-oxo-3h-indol-3-ylidene)methyl)-2,4-dimethyl-1h-pyrrole-3-propanoic acid':ti,ab OR '(z)-3-(2,4-dimethyl-5-(2-oxo-1,2-dihydro-indol-3-ylidenemethyl)-1h-pyrrol-3-yl)-propionic acid':ti,ab OR '(z)-3-(2,4-dimethyl-5-(2-oxo-1,2-dihydro-indol-3-ylidenemethyl)-1h-pyrrol-3-yl)propionic acid':ti,ab OR 'su 6668':ti,ab OR 'tsu-68':ti,ab OR 'su006668':ti OR 'tsu 68':ti,ab OR 'su-6668':ti,ab OR 'su6668':ti,ab | 155 |
| #29 | #27 OR #28 | 1,063 |
| #30 | #3 AND #6 AND #29 | 68 |
| #31 | 'lenvatinib'/exp OR 'lenvatinib' | 5,876 |
| #32 | '4-(3-chloro-4-(n-cyclopropylureido)phenoxy)-7-methoxyquinoline-6-carboxamide':ti,ab OR '4-(3-chloro-4-((cyclopropylaminocarbonyl)amino)phenoxy)-7-methoxy-6-quinolinecarboxamide':ti,ab OR 'lenvima':ti,ab OR 'e 7080':ti,ab OR 'e-7080':ti,ab OR 'er-203492-00':ti,ab OR 'e7080':ti,ab OR 'e-7080 mesylate':ti,ab OR 'e7080 mesylate':ti,ab OR 'lenvatinib metabolite m2':ti,ab OR '4-(3-chloro-4-(((cyclopropylamino)carbonyl)amino)phenoxy)-7-hydroxy-6-quinolinecarboxamide':ti,ab OR 'lenvatinib mesylate':ti,ab OR 'lenvatinib methanesulfonate':ti,ab OR 'n-(4-((6-carbamoyl-7-methoxyquinolin-4-yl)oxy)-2-chlorophenyl)-n-cyclopropylurea monomethanesulfonate':ti,ab OR 'lenvatinib mesilate':ti,ab | 199 |
| #33 | #31 OR #32 | 5,900 |
| #34 | #3 AND #6 AND #33 | 655 |
|  | Search strategy in Cochrane |  |
| No | Query | Results |
| #1 | MeSH descriptor: [Liver Neoplasms] explode all trees | 3,835 |
| #2 | (Neoplasms, Hepatic):ti,ab,kw OR (Neoplasms, Liver):ti,ab,kw OR (Liver Neoplasm):ti,ab,kw OR (Neoplasm, Liver):ti,ab,kw OR (Hepatic Neoplasms):ti,ab,kw | 7,251 |
| #3 | (Hepatic Neoplasm):ti,ab,kw OR (Neoplasm, Hepatic):ti,ab,kw OR (Cancer of Liver):ti,ab,kw OR (Hepatocellular Cancer):ti,ab,kw OR (Cancers, Hepatocellular):ti,ab,kw | 14,323 |
| #4 | (Hepatocellular Cancers):ti,ab,kw OR (Hepatic Cancer):ti,ab,kw OR (Cancer, Hepatic):ti,ab,kw OR (Cancers, Hepatic):ti,ab,kw OR (Hepatic Cancers):ti,ab,kw | 6,815 |
| #5 | (Liver Cancer):ti,ab,kw OR (Cancer, Liver):ti,ab,kw OR (Cancers, Liver):ti,ab,kw OR (Liver Cancers):ti,ab,kw OR (Cancer of the Liver):ti,ab,kw | 12,570 |
| #6 | (Cancer, Hepatocellular):ti,ab,kw | 2,752 |
| #7 | #1 or #2 or #3 or #4 or #5 or #6 | 17,673 |
| #8 | MeSH descriptor: [Chemoembolization, Therapeutic] explode all trees | 392 |
| #9 | (Therapeutic Chemoembolization):ti,ab,kw OR (Chemoembolizations, Therapeutic):ti,ab,kw OR (Therapeutic Chemoembolizations):ti,ab,kw | 576 |
| #10 | #8 or #9 | 576 |
| #11 | MeSH descriptor: [Sorafenib] explode all trees | 623 |
| #12 | (Nexavar):ti,ab,kw OR (Sorafenib N Oxide):ti,ab,kw OR (BAY 43 9006):ti,ab,kw OR (BAY 439006):ti,ab,kw OR (Sorafenib N-Oxide):ti,ab,kw | 176 |
| #13 | (BAY-673472):ti,ab,kw OR (BAY 673472):ti,ab,kw OR (BAY5459085):ti,ab,kw OR (BAY 545 9085):ti,ab,kw OR (BAY 5459085):ti,ab,kw | 0 |
| #14 | (Sorafenib Tosylate):ti,ab,kw | 42 |
| #15 | #11 or #12 or #13 or #14 | 779 |
| #16 | #7 and #10 and #15 | 40 |
| #17 | MeSH descriptor: [Sunitinib] explode all trees | 418 |
| #18 | (SU 011248):ti,ab,kw OR (Sunitinib Malate):ti,ab,kw OR (Sutent):ti,ab,kw OR (SU 11248):ti,ab,kw OR (SU011248):ti,ab,kw | 184 |
| #19 | #17 or #18 | 542 |
| #20 | #7 and #10 and #19 | 2 |

**Supplementary Table 2. Characteristics of included studies in this analysis.**

| study | Year | Region | Design | Group | Sample size | Age  (years) | Gender  (M/F) | BCLC  (A/B/C) | Vascular invasion(yes/no) | EHS  (yes/no) |
| --- | --- | --- | --- | --- | --- | --- | --- | --- | --- | --- |
| Kuang  (11) | 2021 | China | Cohrot study | Sorafenib+TACE  TACE | 66  66 | 60.46±9.91  58.97±10.52 | 49/17  55/11 | 0/29/37  0/24/42 | 17/49  21/45 | 11/55  8/58 |
| Koch  (12) | 2021 | German | Cohort study | Sorafenib+TACE  TACE | 54  65 | 64.0(34-77)  67.0(41-80) | 47/7  53/12 | 0/0/54  0/0/65 | 18/36  25/40 | 22/32  22/43 |
| Zou  (13) | 2021 | China | Cohort study | Sorafenib+TACE  TACE | 42  43 | 58.31±7.83  58.83±8.11 | 32/10  31/12 | 0/23/19  0/25/18 | NA  NA | 4/38  3/40 |
| Cao  (14) | 2020 | China | Cohort study | Sorafenib+TACE  TACE | 50  50 | NA  NA | NA  NA | NA  NA | NA  NA | NA  NA |
| Liu  (15) | 2020 | China | Cohort  study | Sorafenib+TACE  TACE | 59  59 | 56.31±9.87  58.11±10.44 | 37/22  32/27 | 0/30/29  0/36/23 | NA  NA | NA  NA |
| Liu  (16) | 2020 | China | Cohort study | Sorafenib+TACE  TACE | 35  40 | 68.24(35-88)  56(35-85) | 30/5  32/8 | 0/0/35  0/0/40 | 24/11  28/12 | 11/24  18/22 |
| Wang  (17) | 2020 | China | Cohort study | Sorafenib+TACE  TACE | 313  1406 | 53.7±12.0  56.7±12.1 | 267/46  1183/223 | 36/167/110  192/756/458 | NA  NA | NA  NA |
| Kudo  (18) | 2019 | Japan | RCT | Sorafenib+TACE  TACE | 80  76 | 72.0(36-85)  73.0(53-86) | 63/17  55/21 | 27/44/9  33/34/9 | 0/80  0/76 | 0/80  0/76 |
| Ren  (19) | 2019 | China | Cohort study | Sorafenib+TACE  TACE | 61  247 | <60:39; ≥60:22  <50:148; ≥50:99 | 48/13  209/38 | 0/30/31  0/150/97 | 20/41  71/176 | 14/47  15/232 |
| Lei  (20) | 2018 | China | Cohort study | Sorafenib+TACE  TACE | 38  29 | 52±5  51±6 | 24/14  18/11 | 0/38/0  0/29/0 | NA  NA | NA  NA |
| Meyer  (21) | 2017 | UK | RCT | Sorafenib+TACE  TACE | 157  156 | 65(57-71)  68(63-74) | 139/18  138/18 | NA  NA | NA  NA | NA  NA |
| Lencioni  (22) | 2016 | USA | RCT | Sorafenib+TACE  TACE | 154  153 | 64.5  63.0 | 135/19  126/27 | NA  NA | 0/154  0/153 | 0/154  0/153 |
| Wan  (23) | 2016 | China | Cohort  study | Sorafenib+TACE  TACE | 245  245 | <50:115; ≥50;130  <50:115; ≥50;130 | 218/27  218/27 | NA  NA | 70/175  27/218 | 43/202  36/209 |
| Hu  (24) | 2014 | China | Cohort study | Sorafenib+TACE  TACE | 82  198 | 61±11  57±12 | 69/13  159/39 | NA  NA | NA  NA | 27/55  60/138 |
| Zhu  (25) | 2014 | China | Cohort study | Sorafenib+TACE  TACE | 46  45 | 48.4±8.1  51.9±12.2 | 39/7  38/7 | NA  NA | NA  NA | 17/29  18/27 |
| Bai  (26) | 2013 | China | Cohort  study | Sorafenib+TACE  TACE | 82  222 | 54±13  50±12 | 73/9  188/34 | 0/19/63  0/81/141 | 11/71  20/202 | 24/58  58/164 |
| Muhammad  (27) | 2013 | USA | Cohort study | Sorafenib+TACE  TACE | 13  30 | 61.4±7.5  59.2±7.4 | NA  NA | 6/2/5  22/8/0 | NA  NA | NA  NA |
| Qu  (28) | 2012 | China | Cohort study | Sorafenib+TACE  TACE | 45  45 | 51±11.7  49±11.0 | 41/4  41/4 | 0/16/29  0/17/28 | 20/25  23/22 | 11/34  12/33 |
| Kudo  (29) | 2011 | Japan | RCT | Sorafenib+TACE  TACE | 229  229 | 69  70 | 174/55  168/61 | NA  NA | 0/229  0/229 | 0/229  0/229 |
| Chen  (30) | 2022 | China | Cohort study | Lenvatinib+TACE  TACE | 39  148 | 59.1±11.8  59.7±10.8 | 38/1  116/32 | NA  NA | 13/26  44/104 | NA  NA |
| Xie  (31) | 2022 | China | Cohort study | Lenvatinib+TACE  TACE | 51  53 | 56.83±5.68  56.59±5.74 | 38/13  41/12 | 0/30/21  0/34/19 | NA  NA | NA  NA |
| Fu  (32) | 2021 | China | Cohort  study | Lenvatinib+TACE  TACE | 60  60 | 60(25-76)  60(33-81) | 10/50  5/55 | 2/33/25  3/26/31 | 21/39  39/21 | 9/51  9/51 |
| Li  (33) | 2022 | China | Cohort study | Apatinib+TACE  TACE | 39  34 | 60.4±8.5  58.3±8.5 | 36/3  28/6 | 0/23/16  0/14/20 | 17/22  20/14 | NA  NA |
| Li  (34) | 2022 | China | Cohort study | Apatinib+TACE  TACE | 45  51 | 56.8±8.3  59.5±8.9 | 38/7  47/4 | 0/26/19  0/35/16 | 25/20  19/32 | NA  NA |
| Wang  (35) | 2022 | China | Cohort study | Apatinib+TACE  TACE | 119  49 | 54.4±10.1  57.9±11.9 | 104/15  40/9 | 0/73/46  0/29/20 | 45/74  20/29 | NA  NA |
| Kan  (36) | 2020 | China | Cohort study | Apatinib+TACE  TACE | 126  97 | 50.5±10.3  53.7±10.3 | 112/14  81/16 | 0/0/126  0/0/97 | 8/118  6/91 | 62/64  50/47 |
| Liu  (37) | 2020 | China | Cohort study | Apatinib+TACE  TACE | 27  34 | 70.23±6.75  71.10±5.90 | 17/10  26/8 | 0/22/5  0/27/7 | 3/24  5/29 | NA  NA |
| Shen  （38） | 2020 | China | Cohort study | Apatinib+TACE  TACE | 40  280 | <50:17;≥50:23  <50:134;≥50:146 | 38/2  265/15 | NA  NA | 8/32  63/217 | 13/27  62/218 |
| Liu  （39） | 2019 | China | Cohort study | Apatinib+TACE  TACE | 34  48 | 53.3 9.4  56.5 9.7 | 29/5  39/9 | 0/18/16  0/28/20 | 16/18  21/37 | 5/29  8/40 |
| Fan  （40） | 2019 | China | Cohort study | Apatinib+TACE  TACE | 85  103 | 49(17-71)  50(19-80) | 68/17  71/32 | NA  NA | 85/0  103/0 | NA  NA |
| Chen  （41） | 2018 | China | Cohort study | Apatinib+TACE  TACE | 27  53 | 45.8±11.0  54.4±11.9 | 23/4  43/10 | 0/0/27  0/0/53 | NA  NA | 9/18  16/37 |
| Yang  （42） | 2018 | China | Cohort study | Apatinib+TACE  TACE | 25  22 | ≤65:13;>65:12  ≤65:12;>65:10 | 20/5  18/4 | 0/10/15  0/10/12 | NA  NA | NA  NA |
| Lu  (43) | 2017 | China | RCT | Apatinib+TACE  TACE | 20  22 | 56.1±10.79  58.9±9.38 | 16/4  17/5 | 0/18/2  0/19/3 | NA  NA | NA  NA |
| Guo  (44) | 2020 | China | Cohort study | Anlotinib+TACE  TACE | 36  46 | 65.41±12.44  56.28±11.66 | 29/7  38/8 | 3/16/17  1/23/22 | 12/24  20/26 | 8/28  14/32 |
| Kudo  (45) | 2014 | Asia, Europe, USA | RCT | Brivanib+TACE  TACE | 249  253 | 57(21-85)  59(25-85) | 206/43  216/37 | 65/129/55  57/150/46 | 0/249  0/253 | 0/249  0/253 |
| Turpin  (46) | 2020 | France | RCT | Sunitinib+TACE  TACE | 39  39 | 66.0(46.0-84.7)  67.4(43.7-84.7) | 36/3  35/4 | NA/33/NA  NA/25/NA | NA  NA | NA  NA |
| Chen  (47) | 2014 | China | Cohort study | Sunitinib+TACE  TACE | 38  65 | 59(35-74)  56(31-78) | 31/7  54/11 | 0/0/38  0/0/65 | 32/6  48/17 | NA  NA |
| Hidaka  (48) | 2019 | Japan | RCT | Orantinib+TACE  TACE | 219  213 | 71.0(44-86)  71.0(46-87) | 178/41  176/37 | 71/121/27  64/119/30 | NA  NA | NA  NA |
| Kudo  (49) | 2017 | Japan, South  Korea, Taiwan | RCT | Orantinib+TACE  TACE | 444  444 | 66.2±10.2  65.4±10.0 | 363/81  364/80 | 158/209/74  135/229/72 | 34/410  37/407 | 0/444  0/444 |
| Inaba  (50) | 2013 | Japan | RCT | Orantinib+TACE  TACE | 50  51 | ≤65:39;>65:11  ≤65:42;>65:9 | 39/11  43/8 | 21/24/5  21/27/2 | NA  NA | 0/50  0/51 |
| Ding  (51) | 2021 | China | Cohort study | Lenvatinib+TACE  Sorafenib+TACE | 32  32 | 57±11  56±11 | 52/12  25/7 | NA  NA | 32/0  32/0 | 13/19  9/23 |
| Yang  (52) | 2021 | China | Cohort study | Lenvatinib+TACE  Sorafenib+TACE | 59  57 | 54.05±11.35  56.18±12.16 | 54/5  50/7 | NA  NA | 59/0  57/0 | 0/59  0/57 |
| Cao  (53) | 2021 | China | Cohort study | Sorafenib+TACE  Apatinib+TACE | 32  41 | 52.7±12.4  51.6±9.6 | 28/4  37/4 | NA  NA | 32/0  41/0 | 18/14  24/17 |
| Qiu  (54) | 2021 | China | Cohort study | Sorafenib+TACE  Apatinib+TACE | 115  86 | <52:53; ≥52:62  <52:42; ≥52:44 | 104/11  78/8 | NA  NA | 71/44  55/31 | 66/49  50/36 |
| Xu  (55) | 2018 | China | Cohort study | Sunitinib+TACE  Sorafenib+TACE | 51  53 | 60(41-71)  62(45-75) | 42/9  41/12 | 6/11/34  5/16/32 | 14/37  19/34 | 51/0  53/0 |

**Supplementary Table 3. Quality evaluation of included literature.**

| Cohort studies | Selection | | | | Comparability | Exposure/Outcome | | | Total |
| --- | --- | --- | --- | --- | --- | --- | --- | --- | --- |
|  | 1^a^ | 2^a^ | 3^a^ | 4^a^ | 5^a^ | 6^a^ | 7^a^ | 8^a^ |  |
| Kuang, 2021 (11) | * | * | * | * | ** | * |  | * | 8 |
| Coch, 2021 (12) | * | * | * | * | ** | * | * | * | 9 |
| Zou, 2021 (13) | * | * | * | * | ** | * | * | * | 9 |
| Cao, 2020 (14) | * | * | * | * | ** | * | * | * | 9 |
| Liu, 2020 (15) | * | * | * | * | ** | * | * | * | 9 |
| Liu, 2020 (16) | * | * | * | * | ** | * | * | * | 9 |
| Wang, 2020 (17) | * | * | * | * | ** | * | * | * | 9 |
| Ren, 2019 (19) | * | * | * |  | ** | * | * | * | 8 |
| Lei, 2018 (20) | * | * | * | * | ** | * | * | * | 9 |
| Wan, 2016 (23) | * | * | * | * | ** | * | * | * | 9 |
| Hu, 2014 (24) | * | * | * |  | ** | * | * | * | 8 |
| Zhu, 2014 (25) | * | * | * | * | ** | * | * | * | 9 |
| Bai, 2013 (26) | * | * | * | * | ** | * | * | * | 9 |
| Muhammad, 2013 (27) | * | * | * |  | ** | * | * | * | 8 |
| Qu, 2012 (28) | * | * | * | * | ** | * | * | * | 9 |
| Chen, 2022 (30) | * | * | * |  | ** | * | * | * | 8 |
| Xie,2022 (31) | * | * | * | * | ** | * | * | * | 9 |
| Fu, 2021 (32) | * | * | * |  | ** | * | * | * | 8 |
| Li, 2022 (33) | * | * | * | * | ** | * | * | * | 9 |
| Li, 2022 (34) | * | * |  |  | ** | * | * | * | 7 |
| Wang, 2022 (35) | * | * | * | * | ** | * | * | * | 9 |
| Kan, 2020 (36) | * | * | * | * | ** | * | * | * | 9 |
| Liu, 2020 (37) | * | * | * | * | ** | * | * | * | 9 |
| Shen, 2020 (38) | * | * | * |  | ** | * | * | * | 8 |
| Liu, 2019 (39) | * | * | * | * | ** | * | * | * | 9 |
| Fan, 2019 (40) | * | * | * | * | ** | * | * | * | 9 |
| Chen, 2018 (41) | * | * | * |  | ** | * |  | * | 7 |
| Yang, 2018 (42) | * |  | * | * | ** | * | * | * | 8 |
| Guo, 2020 (44) | * | * | * | * | * | * | * | * | 8 |
| Chen, 2014 (47) | * | * | * | * | ** | * | * | * | 9 |
| Ding, 2021 (51) | * | * | * |  | ** | * | * | * | 8 |
| Yang, 2021 (52) | * | * | * | * | ** | * | * | * | 9 |
| Cao, 2021 (53) | * | * | * |  | ** | * | * | * | 8 |
| Qiu, 2021 (54) | * | * | * |  | ** | * | * | * | 8 |
| Xu, 2018 (55) | * | * | * | * | ** | * | * | * | 9 |
| RCTs | 1^b^ | 2^b^ | 3^b^ | 4^b^ | 5^b^ | 6^b^ | 7^b^ |  |  |
| Kudo, 2019 (18) | L | L | U | U | L | L | L |  |  |
| Meyer, 2017 (21) | L | L | L | L | L | L | L |  |  |
| Lencioni, 2016 (22) | L | L | U | U | L | L | L |  |  |
| Kudo, 2011 (29) | L | L | U | U | L | L | L |  |  |
| Lu, 2017 (43) | L | L | L | U | L | L | L |  |  |
| Kudo, 2014 (45) | L | U | H | L | L | L | L |  |  |
| Turpin, 2020 (46) | L | L | L | L | L | L | L |  |  |
| Hidaka, 2019 (48) | L | U | H | L | L | L | L |  |  |
| Kudo, 2017 (49) | L | U | H | L | L | L | L |  |  |
| Inaba, 2013 (50) | L | L | U | L | L | L | L |  |  |

1^a^, the representativeness of the exposure cohort; 2^a^, the selection of the non-exposed cohort;3^a^, the determination of the exposure; 4^a^, the absence of the disease to be studied at the beginning of the study; 5^a^, the comparability of the exposure cohort and the non-exposed cohort; 6^a^, the measurement method of the results; 7^a^, whether the follow-up time was long enough; 8^a^, the integrity of the follow-up. 1^b^, random sequence generation; 2^b^, allocation concealment; 3^b^, implementation bias; 4^b^, measurement bias; 5^b^, follow-up bias; 6^b^, reporting bias; 7^b^, other biases. L, low risk of bias; H, high risk of bias; U, unclear risk of bias.

**Supplementary Table 4. Analysis of treatment ranking probability in patients with uHCC.**

| **Intervention** | **Rank 1** | **Rank 2** | **Rank 3** | **Rank 4** | **Rank 5** | **Rank 6** | **Rank 7** | **Rank 8** | |
| --- | --- | --- | --- | --- | --- | --- | --- | --- | --- |
| **OS** |  |  |  |  |  |  |  |  | |
| Anlo+TACE | 0.0867625 | 0.1354 | 0.172175 | 0.1828625 | 0.17075 | 0.1043 | 0.065425 | 0.082325 | |
| Apat+TACE | 0.62235 | 0.305775 | 0.0631375 | 0.007525 | 0.0011125 | 0.0001 | 0 | 0 | |
| Briv+TACE | 0.018675 | 0.05135 | 0.0972375 | 0.15035 | 0.22345 | 0.1824125 | 0.1117875 | 0.1647375 | |
| Lenv+TACE | 0.2680125 | 0.4064125 | 0.1905375 | 0.0850625 | 0.0368375 | 0.0113125 | 0.0013 | 0.000525 | |
| Oran+TACE | 0.0000125 | 0.000425 | 0.00265 | 0.0211875 | 0.077 | 0.1568875 | 0.247725 | 0.4941125 | |
| Sora+TACE | 0.0008 | 0.07505 | 0.4037875 | 0.37785 | 0.1264625 | 0.0158375 | 0.0002125 | 0 | |
| Suni+TACE | 0.0033875 | 0.0255875 | 0.0704625 | 0.169 | 0.2913625 | 0.2339625 | 0.110225 | 0.0960125 | |
| TACE | 0 | 0 | 0.0000125 | 0.0061625 | 0.073025 | 0.2951875 | 0.463325 | 0.1622875 | |
| **PFS** |  |  |  |  |  |  |  |  | |
| Anlo+TACE | 0.0117125 | 0.0528625 | 0.1991375 | 0.3267625 | 0.1858875 | 0.1259375 | 0.0977 |  | |
| Apat+TACE | 0.125875 | 0.5814 | 0.246125 | 0.0398875 | 0.005525 | 0.00115 | 0.0000375 |  | |
| Lenv+TACE | 0.7885375 | 0.138475 | 0.051775 | 0.015275 | 0.0044 | 0.001225 | 0.0003125 |  | |
| Oran+TACE | 0.0705625 | 0.2035125 | 0.3437875 | 0.1708875 | 0.0952375 | 0.0612625 | 0.05475 |  | |
| Sora+TACE | 0.0002625 | 0.0044625 | 0.075 | 0.274125 | 0.4234625 | 0.212925 | 0.0097625 |  | |
| Suni+TACE | 0.00305 | 0.0192875 | 0.0839625 | 0.1663625 | 0.2089125 | 0.212825 | 0.3056 |  | |
| TACE | 0 | 0 | 0.0002125 | 0.0067 | 0.076575 | 0.384675 | 0.5318375 |  | |
| **ORR** |  |  |  |  |  |  |  |  | |
| Anlo+TACE | 0.0149125 | 0.0140625 | 0.0277 | 0.0794875 | 0.1009375 | 0.1662 | 0.5967 |  | |
| Apat+TACE | 0.4471125 | 0.3454 | 0.163175 | 0.0399125 | 0.0042 | 2.00E-04 | 0 |  | |
| Briv+TACE | 0.089425 | 0.061225 | 0.10345 | 0.2661875 | 0.161625 | 0.18105 | 0.1370375 |  | |
| Lenv+TACE | 0.2528125 | 0.198925 | 0.3070125 | 0.1698 | 0.0492125 | 0.016925 | 0.0053125 |  | |
| Sora+TACE | 0.1866875 | 0.3657375 | 0.3436375 | 0.0946875 | 0.0088375 | 0.0004125 | 0 |  | |
| Suni+TACE | 0.00905 | 0.01465 | 0.0504125 | 0.1865625 | 0.232975 | 0.30955 | 0.1968 |  | |
| TACE | 0 | 0 | 0.0046125 | 0.1633625 | 0.4422125 | 0.3256625 | 0.06415 |  | |
| **DCR** |  |  |  |  |  |  |  |  | |
| Anlo+TACE | 0.1790375 | 0.08965 | 0.10525625 | 0.16576875 | 0.13859375 | 0.133875 | 0.18781875 |  | |
| Apat+TACE | 0.455575 | 0.32051875 | 0.1584 | 0.05380625 | 0.010775 | 0.00091875 | 0.00000625 |  | |
| Briv+TACE | 0.07399375 | 0.053525 | 0.0713875 | 0.12680625 | 0.149925 | 0.16445 | 0.3599125 |  | |
| Lenv+TACE | 0.1458125 | 0.17023125 | 0.26129375 | 0.24144375 | 0.12023125 | 0.04483125 | 0.01615625 |  | |
| Sora+TACE | 0.1281375 | 0.33708125 | 0.33291875 | 0.161825 | 0.0367 | 0.003275 | 0.0000625 |  | |
| Suni+TACE | 0.01744375 | 0.0289875 | 0.0660125 | 0.162075 | 0.22188125 | 0.2401125 | 0.2634875 |  | |
| TACE | 0 | 0.00000625 | 0.00473125 | 0.088275 | 0.32189375 | 0.4125375 | 0.17255625 |  | |
| **G3-AEs** |  |  |  |  |  |  |  |  | |
| Apat+TACE | 0.0138125 | 0.0512375 | 0.0539 | 0.084575 | 0.108425 | 0.68805 |  |  | |
| Briv+TACE | 0.0457375 | 0.102725 | 0.168675 | 0.2296625 | 0.2971875 | 0.1560125 |  |  |  |
| Lenv+TACE | 0.02785 | 0.127325 | 0.33025 | 0.2486125 | 0.22625 | 0.0397125 |  |  |  |
| Oran+TACE | 0.0224625 | 0.11145 | 0.209625 | 0.2801375 | 0.2655125 | 0.1108125 |  |  |  |
| Sora+TACE | 0.40755 | 0.2726375 | 0.1215375 | 0.1036625 | 0.090475 | 0.0041375 |  |  |  |
| TACE | 0.4825875 | 0.334625 | 0.1160125 | 0.05335 | 0.01215 | 0.001275 |  |  |  |
| **AEs** |  |  |  |  |  |  |  |  |  |
| Briv+TACE | 0.2813 | 0.221875 | 0.3708125 | 0.065475 | 0.0605375 |  |  |  |  |
| Lenv+TACE | 0.046875 | 0.04125 | 0.0699625 | 0.3079375 | 0.533975 |  |  |  |  |
| Oran+TACE | 0.3428375 | 0.2637125 | 0.2799625 | 0.060975 | 0.0525125 |  |  |  |  |
| Sora+TACE | 0.012375 | 0.0311375 | 0.076275 | 0.534025 | 0.3461875 |  |  |  |  |
| TACE | 0.3166125 | 0.442025 | 0.2029875 | 0.0315875 | 0.0067875 |  |  |  |  |

**Supplementary Table 5. Node-splitting method for evaluating consistency between direct and indirect comparison.**

| Treatment comparisons | Direct effect | Indirect effect | Overall | P value of node-splitting method |
| --- | --- | --- | --- | --- |
| OS |  |  |  |  |
| Sora+TACE vs Apat+TACE | 1.1 (0.86, 1.4) | 1.5 (1.1, 1.9) | 1.2 (1.0, 1.5) | 0.08 |
| TACE vs Apat+TACE | 1.8 (1.5, 2.3) | 1.3 (1.1, 1.8) | 1.6 (1.3, 2.0) | 0.07 |
| Sora+TACE vs Lenv+TACE | 1.7 (1.1, 2.6) | 0.93 (0.67, 1.3) | 1.1 (0.88, 1.5) | 0.04 |
| TACE vs Lenv+TACE | 1.2 (0.9, 1.7) | 2.1 (1.4, 3.4) | 1.5 (1.1, 2.0) | 0.04 |
| Suni+TACE vs Sora+TACE | 1.6 (0.9, 2.9) | 1.1 (0.79, 1.5) | 1.2 (0.89, 1.6) | 0.21 |
| TACE vs Sora+TACE | 1.3 (1.1, 1.5) | 1.4 (1.1, 1.9) | 1.3 (1.2, 1.5) | 0.40 |
| TACE vs Suni+TACE | 1.2 (0.9, 1.6) | 0.79 (0.44, 1.4) | 1.1 (0.85, 1.5) | 0.22 |
| PFS |  |  |  |  |
| Sora+TACE vs Apat+TACE | 1.5 (0.87, 2.7) | 1.4 (0.98, 1.9) | 1.5 (1.1, 1.9) | 0.82 |
| TACE vs Apat+TACE | 1.7 (1.3, 2.2) | 1.8 (1.0, 3.5) | 1.7 (1.3, 2.2) | 0.81 |
| Sora+TACE vs Lenv+TACE | 2.1 (0.92, 4.6) | 1.8 (0.92, 3.3) | 1.9 (1.1, 3.0) | 0.75 |
| TACE vs Lenv+TACE | 2.1 (1.1, 3.8) | 2.4 (1.1, 5.8) | 2.2 (1.4, 3.6) | 0.76 |
| Suni+TACE vs Sora+TACE | 1.6 (0.95, 2.7) | 0.73 (0.44, 1.2) | 1.1 (0.72, 1.7) | 0.04 |
| TACE vs Sora+TACE | 1.1 (0.98, 1.5) | 1.4 (0.89, 2.1) | 1.2 (1.0, 1.5) | 0.42 |
| TACE vs Suni+TACE | 1.5 (0.93, 2.5) | 0.69 (0.40, 1.2) | 1.1 (0.73, 1.6) | 0.04 |
| ORR |  |  |  |  |
| Sora+TACE vs Apat+TACE | 1.8 (0.80, 4.3) | 0.66 (0.42, 1.1) | 0.86 (0.56, 1.4) | 0.04 |
| TACE vs Apat+TACE | 0.37 (0.27, 0.51) | 1.0 (0.42, 2.5) | 0.42 (0.30, 0.58) | 0.04 |
| Suni+TACE vs Sora+TACE | 0.49 (0.15, 1.7) | 0.40 (0.14, 1.1) | 0.44 (0.20, 0.94) | 0.79 |
| TACE vs Sora+TACE | 0.55 (0.37, 0.78) | 0.27 (0.12, 0.59) | 0.48 (0.33, 0.67) | 0.11 |
| TACE vs Suni+TACE | 1.2 (0.44, 3.2) | 0.97 (0.27, 3.4) | 1.1 (0.51, 2.3) | 0.79 |
| DCR |  |  |  |  |
| Sora+TACE vs Apat+TACE | 1.2 (0.78, 1.8) | 0.86 (0.67, 1.1) | 0.93 (0.75, 1.2) | 0.16 |
| TACE vs Apat+TACE | 0.67 (0.50, 0.80) | 0.95 (0.59, 1.5) | 0.70 (0.59, 0.82) | 0.16 |
| Sora+TACE vs Lenv+TACE | 0.72 (0.39, 1.3) | 1.2 (0.84, 1.8) | 1.1 (0.76, 1.5) | 0.14 |
| TACE vs Lenv+TACE | 0.88 (0.64, 1.2) | 0.53 (0.28, 0.98) | 0.79 (0.58, 1.1) | 0.14 |
| Suni+ATCE vs Sora+TACE | 0.80 (0.43, 1.5) | 0.73 (0.41, 1.2) | 0.76 (0.51, 1.1) | 0.80 |
| TACE vs Sora+TACE | 0.76 (0.62, 0.91) | 0.74 (0.51, 1.1) | 0.75 (0.63, 0.88) | 0.88 |
| TACE vs Suni+TACE | 1.0 (0.65, 1.7) | 0.93 (0.48, 1.8) | 0.99 (0.69, 1.5) | 0.81 |
